# Phytochemical and Biological Investigations of Crude Extracts of *Astragalus pisidicus*

**DOI:** 10.3390/ph18010010

**Published:** 2024-12-25

**Authors:** Esra Aydemir, Elif Odabaş Köse, Serap Özkaya Gül, Alaaddin Korkut, A. Cansu Kilit, Mehmet Engin Celep, Mustafa Yavuz, R. Süleyman Göktürk, Cengiz Sarikurkcu

**Affiliations:** 1 Department of Biology, Faculty of Science, Akdeniz University, TR-07058 Antalya, Turkey; 202151006001@ogr.akdeniz.edu.tr (S.Ö.G.); 202451006003@ogr.akdeniz.edu.tr (A.K.); myavuz@akdeniz.edu.tr (M.Y.); gokturk@akdeniz.edu.tr (R.S.G.); 2 Medical Laboratory Program, Vocational School of Health Services, Akdeniz University, TR-07058 Antalya, Turkey; elifkose@akdeniz.edu.tr; 3 Biomedical Device Technology Program, Department of Electronics and Automation, Technical Sciences Vocational School, Akdeniz University, TR-07058 Antalya, Turkey; acansukilit@akdeniz.edu.tr; 4 Department of Pharmacognosy, Faculty of Pharmacy, Yeditepe University, Atasehir, TR-34755 Istanbul, Turkey; ecelep@yeditepe.edu.tr; 5 Department of Analytical Chemistry, Faculty of Pharmacy, Afyonkarahisar Health Sciences University, TR-03100 Afyonkarahisar, Turkey; cengiz.sarikurkcu@afsu.edu.tr

**Keywords:** apoptosis, *Astragalus*, antimicrobial, antioxidant, cytotoxicity

## Abstract

**Background/Objectives**: *Astragalus* L. is a genus of the Fabaceae family, encompassing over 3000 species globally, with 380 species found in Turkey. This is the inaugural examination of the phytochemical, antioxidant, antibacterial, and cytotoxic properties of *Astragalus pisidicus*. **Methods**: The water and methanolic fractions of four parts (stems, flowers, leaves, root) as well as the whole plant were quantified and identified by Liquid Chromatography Electrospray Ionization Tandem Mass Spectrometry (LC–ESI–MS/MS) analysis. Cell death was assessed using the WST-1 assay, while apoptosis was identified by colorimetric protease assay for caspase 2, -3, -6, -8, and -9, as well as cellular DNA fragmentation assay. Antioxidant activity of *A. pisidicus* water and methanolic extracts was investigated with eight different assays. Antimicrobial activities of the extracts were evaluated against 16 bacterial strains by disc diffusion and broth microdilution methods. **Results**: A total of 13 phytochemicals were detected in the extracts at various concentrations. Hesperidin (147–40,174 µg/g extract) and hyperoside (363–2677 µg/g extract) comprised the principal constituents among the extracts. Fm (IC_50_ = 9.57 µg/mL), Rm (IC_50_ = 14.89 µg/mL), and Sm (IC_50_ = 9.57 µg/mL) were evaluated as active crude extracts on H1299, HT-29, and Panc-1 cells, while Rm (IC_50_ = 32.057 µg/mL) and Fm (IC_50_ = 64.25 µg/mL) were assessed as moderately active on MCF-7 and 22RV1 cells, respectively. The elevation of caspase 2, 3, 6, 8, and 9 enzyme activities, along with DNA fragmentation, signifies that the mode of cell death is apoptosis. According to the disc diffusion test results, Fm, Lm, Sm, and WPm extracts exhibited antimicrobial activity against gram (+) bacteria. **Conclusions**: *A. pisidicus* elicited apoptotic cell death in cancer cells selectively by the activation of caspases and subsequent DNA fragmentation and may serve as a novel source of an apoptosis-inducing anticancer drug.

## 1. Introduction

The genus *Astragalus* L., which is the largest genus of vascular plants in the world, is part of the Papilionoideae subfamily of the Fabaceae (Leguminosae) family. It consists of around 2900 species that are found in various temperate and desert climates, primarily in Europe, Asia, and North America [1]. Turkey is home to a total of 478 different species, which are primarily found in steppes and high mountains. Out of these species, 202 of them are endemic [2].

Since ancient times, many different populations have used extracts from *Astragalus* species to treat a wide variety of diseases. It has been traditionally used in folk medicine as an analgesic (for stomach and gum pain), anticancer (mainly uterine carcinoma and leukemia), antidiarrheal, antiinfective (for sore throat, gum infection, and urinary tract infection), antiinflammatory (for nephritis), antianorexic, antiperspirant, antifatigue, and stimulant, tonic, diuretic, hypoglycemic, laxative, and narcotic agent [3,4,5,6,7,8,9,10,11]. *Radix Astragali*, known as Huangqi in China, is the most important tonic in Traditional Chinese Medicine for strengthening ‘qi’ (vital energy), increasing superficial resistance, and promoting the drainage of pus and the growth of new tissue [12]. *Radix Astragali*, the dried root of *Astragalus membranaceus* (Fisch.) Bge. or *Astragalus membranaceus* (Fisch.) Bge. var. *mongholicus* (Bge.) Hsiao, is typically prepared as a water extract and is used, either alone or together with other medicinal herbs, to treat diabetes, cancer, and cardiovascular, respiratory, and nervous system diseases [13,14].

The *Astragalus* genus is rich in chemical constituents, and much research has been conducted to isolate and purify flavonoids, triterpenoids, polysaccharides, amino acids, alkaloids, ß-sitosterol, metalloids, and anthraquinones. The flavonoids include flavonols, flavones, flavanones, and isoflavonoids, which have been described as having many types of bioactivities. Isoflavones such as calycosin, calycosin-7-glucoside, calycosin-7-*O*-β-d-glucopyranoside, formononetin, and ononin are of significant value due to their important antioxidant, anticancer, anti-inflammatory, anti-infective, and neuroprotective pharmacological effects. Astragalosides I, II, and IV, isoastragalosides I and II are the most abundant saponins isolated from *Astragalus* roots. Among these, astragaloside IV has important pharmacological activities such as antioxidative, antiaging, anti-inflammatory, antiasthma, antidiabetes, and antiatherosclerosis activities, as well as cardioprotective and neuroprotective effects, and for this reason, it has been recognized by the Chinese Pharmacopoeia (2005 version) as a qualitative control biomarker for *A. membranaceus* [3,15]. Astragalus polysaccharide (APS), the main active extract from *A*. *membranaceus*, has been shown to exert multiple pharmacological effects and therefore has high potential in drug development or in the treatment of various diseases [16]. Because of these features, *Astragalus* species have an important potential in the field of health care. Although numerous studies have been carried out worldwide on the genus *Astragalus* and its chemical constituents due to its various activities, there are no data on *Astragalus pisidicus. Astragalus pisidicus* Boiss and Heldr (section: Christina) is an endemic species native to Turkey and is distributed in Central Anatolia and the Western Mediterranean Region [17]. This study will elucidate the phenolic content and cytotoxic, antimicrobial, and antioxidant properties of *A. pisidicus*.

Cancer is a major health problem in the 21st century, affecting society, public health, and the economy [18]. Historically, patients had limited options for cancer treatment, such as surgery, radiation therapy, and chemotherapy, either individually or in combination. While radiation therapy is known to cause harm to healthy cells, tissues, and organs, chemotherapy also carries the potential to damage healthy cells, especially those that undergo rapid reproduction. It is important to mention that every chemotherapy treatment induces harm to healthy cells, although with a decreased mortality rate. An important concern associated with chemotherapy is the emergence of drug resistance. This situation arises when the anti-cancer medication, which initially exhibits a favorable impact on impeding cancer cells, acquires resistance to that treatment. Traditional chemotherapeutic methods suffer from various limitations, including challenges in finding the optimal dosage, reduced selectivity, rapid drug degradation, and the possibility of side effects [19]. Plant-derived natural extracts and/or compounds appear to be a highly appealing substitute for traditional therapies. Since natural compounds offer low toxicity and a lack of adverse reactions, plants yield a multitude of novel cytotoxic compounds each year, opening up new possibilities for cancer treatment [20,21].

Although the discovery and widespread clinical use of antibiotics have contributed to reducing the global burden of infectious diseases, the emergence and spread of antibiotic resistance among clinical pathogens has led to a new crisis [22,23]. The development of new antimicrobial agents is of great importance as a precaution against this increasing threat. Consequently, plant extracts represent a natural source of antimicrobial compounds and potentially offer an effective alternative to the global antibiotic crisis [24]. Plant extracts contain a wide range of secondary compounds that can be utilized in a variety of ways as antimicrobial agents, exhibiting reduced toxicity compared with numerous synthetic antimicrobial agents. Considering that the antimicrobial activity of many plant species has not yet been elucidated, research in this area continues at full speed [25]. The antimicrobial activity of extracts from *Astragalus* species against a variety of bacterial strains has been demonstrated in numerous studies. Additionally, the extracts showed remarkable activity against various gram-positive pathogens such as *Streptococcus pyogenes*, *Staphylococus aureus,* and *Staphylococcus epidermidis* [26,27,28,29].

This study was carried out to identify the main components of *A. pisidicus* from Antalya, Southern Turkey, assess the cytotoxic, antioxidant, and antimicrobial properties of the methanol and water extracts, and delineate the contribution of the extract’s major components towards such biological effects.

## 2. Results

### 2.1. Extraction

The plant materials, i.e., whole plants and the dissected plant parts (root, stem, leaf, and flower parts), were extracted with both water and methanol and then evaporated to dryness under reduced pressure in the rotary evaporator. The yields of the water and methanolic extracts of root, stem, flower, leaf and whole plant of *A. pisidicus* were calculated and represented in Table 1.

### 2.2. Phytochemical Analysis of Extracts

The concentrations of thirty distinct standard phytochemicals in both water and methanol extracts obtained from various parts of *A. pisidicus* were assessed utilizing a previously developed and validated Liquid Chromatography Electrospray Ionization Tandem Mass Spectrometry (LC–ESI–MS/MS) method, and the results are presented in Table 2.

A total of 13 phytochemicals were detected in the extracts at various concentrations. Hesperidin (147–40,174 µg/g extract) and hyperoside (363–2677 µg/g extract) comprised the principal constituents. Moreover, it was found that *p*-hydroxybenzoic acid, protocatechuic acid, and 2,5-dihydroxybenzoic acid were present in significant quantities within the extracts. In contrast, 3,4-dihydroxyphenylacetic acid, pyrocatechol, (−)-epicatechin, caffeic acid, vanillin, verbascoside, sinapic acid, taxifolin, luteolin 7-glycoside, rosmarinic acid, resveratrol, apigenin 7-glycoside, 2-hydroxycinnamic acid, ellagic acid, pinoresinol, eriodictyol, kaempferol, luteolin, and apigenin were not detected in any of the samples.

### 2.3. Cytotoxic Effects of A. pisidicus Extracts

The cytotoxic effects of methanolic and water extracts of *A. pisidicus* on MDAMB-231, MCF-7, 22RV1, A549, Panc-1, HT-29, HeLa, and 293T cell lines were evaluated using the WST-1 assay, which identifies viable cells by the activity of mitochondrial dehydrogenase. Cells were treated with the extracts ranging from 1.95 to 1000 μg/mL for 24, 48, and 72 h of incubation. None of the extracts caused a statistically significant cytotoxic effect on 293T cells throughout any incubation period (*p* > 0.05). The lack of a significant cytotoxic effect of the extracts on 293T cells indicates that they do not exert a direct cytotoxic effect on non-cancerous cells. The extracts demonstrated varying cytotoxic effects across different cancer cells following the incubation durations. Table 3 presents the IC_50_ values for the extracts demonstrating cytotoxic effects on cancer cells within the tested dosage range, together with the corresponding incubation durations. The OD values obtained from the WST-1 test were evaluated using Graph-Pad Prism, version 4.00 (Graph-Pad Software, San Diego, CA), graphed separately for each cell line with SigmaPlot 10, and presented in the Supplementary File (Figures S1–S18).

### 2.4. Colorimetric Protease (Caspase 2, -3, -6, -8, -9) Assay

**Figure 1:**
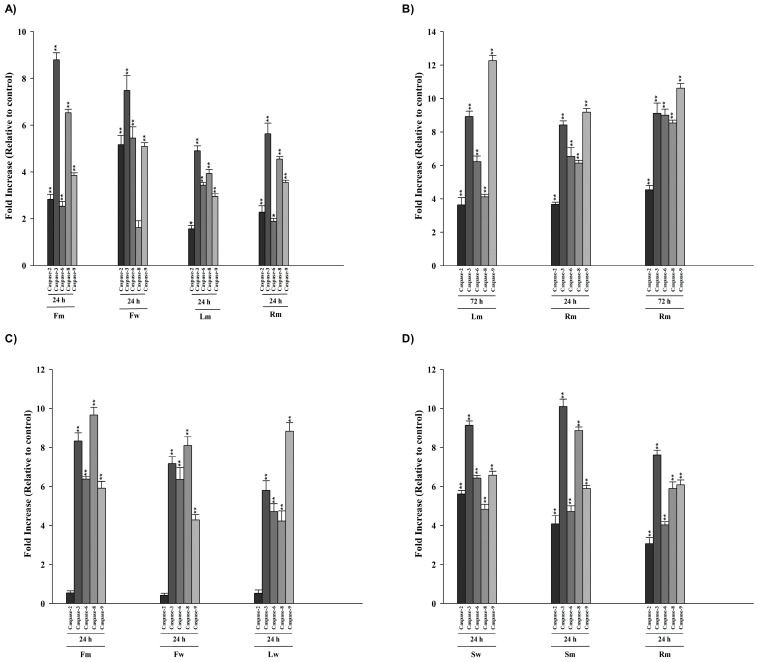
Effect of methanolic and water extracts at IC_50_ concentrations on caspase activities in (**A**) 22RV1; (**B**) A549; (**C**) H1299; and (**D**) PANC1 cells. The student’s *t*-test was used to statistically analyze the data. When compared with the control group, statistically significant differences were defined as * *p* < 0.05, ** *p* < 0.01.

**Figure 2:**
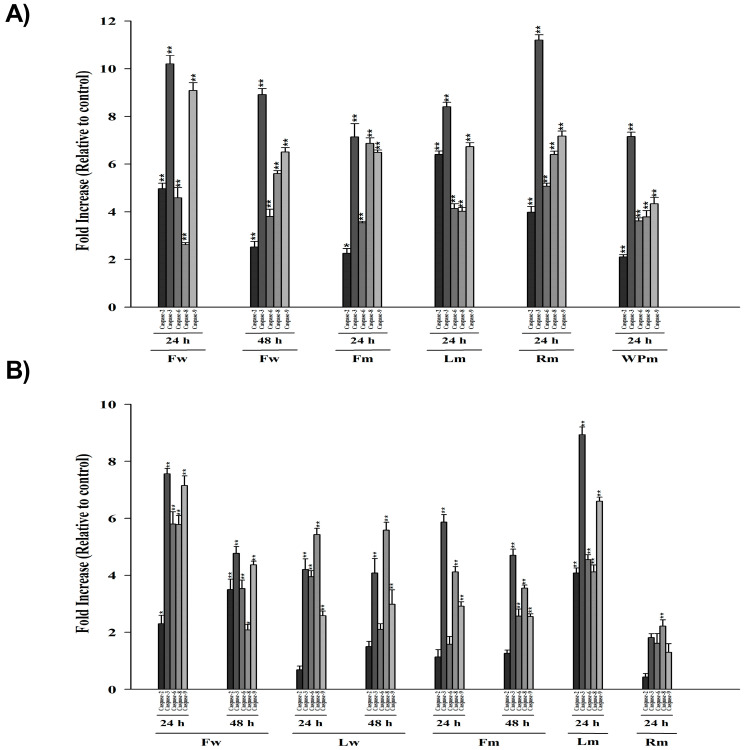
Effect of methanolic and water extracts at IC_50_ concentrations on caspase activities in (**A**) HT29 and (**B**) MCF7 cells. The student’s *t*-test was used to statistically analyze the data. When compared with the control group, statistically significant differences were defined as * *p* < 0.05, ** *p* < 0.01.

The impact of extracts at IC_50_ concentrations for each incubation period on caspases, key proteins that modulate the apoptotic response in cells, was assessed using a colorimetric protease kit. Cells were treated with extracts (¼ IC_50_, ½ IC_50_, IC_50_, 2× IC_50_, 4× IC_50_) for 24 h. Amino acid sequences unique to each caspase—caspase-2 (Ac-VDVAD-pNA), caspase-3 (Ac-DEVD-pNA), caspase-6 (Ac-VEID-pNA), caspase-8 (Ac-IETD-pNA), and caspase-9 (Ac-LEHD-pNA)—were used as substrates. The absorbance values of pNA released from the cleavage of pNA-labeled specific caspase substrates by caspases were quantified at 405 nm. The fold increase in activities of caspase-2, -3, -6, -8, and -9 induced by the extracts at IC_50_ concentrations for each incubation time was determined through direct comparison with the untreated control level (Figure 1 and Figure 2).

### 2.5. Cellular DNA Fragmentation

**Figure 3:**
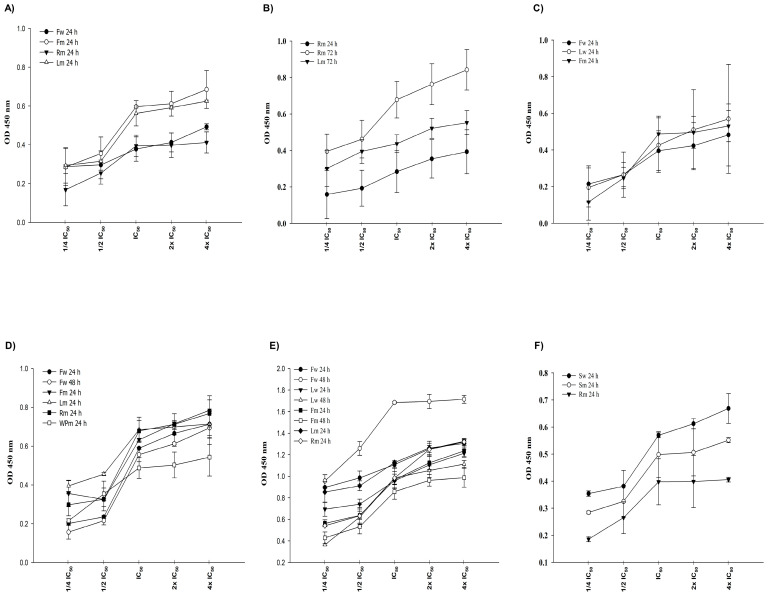
DNA fragmentation induced by water and methanolic extracts in (**A**) 22RV1; (**B**) A549; (**C**) H1299; (**D**) HT29; (**E**) MCF7, and (**F**) PANC1 cells. The Mann–Whitney test was used for statistical analysis of the data, and the results are expressed as mean ± SEM.

An ELISA kit was employed to ascertain if the extracts induce DNA fragmentation in cells at the specified IC_50_ concentrations. The BrdU provided in the kit is a non-radioactive thymidine analog that associates with genomic DNA to assess if the DNA has undergone fragmentation. In order to determine cellular DNA fragmentation, the cells were treated with the extracts prepared in ¼ IC_50_, ½ IC_50_, IC_50_, 2× IC_50_, 4× IC_50_ concentrations for the WST-1 determined incubations. The absorbances of BrdU-labeled DNA fragments were measured at a wavelength of 450 nm (Figure 3). Apoptotic DNA fragmentation is directly proportional to the increased absorbance.

### 2.6. Antimicrobial Activity

The antimicrobial activity of methanol and water extracts of *A. pisidicus* was tested against gram-positive and gram-negative bacteria using the disc diffusion method. According to the results of this test, the water extract was not found to be effective against any bacteria, while the methanol extract was effective against gram (+) bacteria such as *S. aureus*, *Enterococcus faecalis,* and *S. pyogenes*. However, the methanol extract also failed to show any effect against gram-negative bacteria. Table 4 shows the zone of inhibition of the methanol extracts against gram (+) bacteria. The most effective of all tested methanol extracts was WPm extract, while no effect was observed in Rm extract. *S. pyogenes* was the bacterium with the highest inhibition diameters of 12 mm for WPm extract, 11 mm for Fm extract, 10 mm for Sm extract, and 9 mm for Lm extract. The methanol extracts were effective against *S. aureus* species in the range of 8–10 mm inhibition zone. Among these species, *S. aureus* ATCC 25923 showed the highest sensitivity to WPm extract with an inhibition zone of 10 mm. Only WPm extract was effective against *E. faecalis* species with an inhibition zone of 8 mm.

The antimicrobial activity of the methanol and water extracts of *A. pisidicus* was also tested by determining minimum inhibitory concentration (MIC) values. The results of the test showed that WPm extract was the only extract with a MIC value of 8192 µg/mL against *S. pyogenes*. None of the remaining extracts showed any effect against any bacteria (>8192 μg/mL MIC value).

### 2.7. Determination of Antioxidant Activities of Extracts

This study examined the antioxidant activity of *A. pisidicus* water and methanolic extracts using eight different tests. The results regarding total phenolic (TP) and flavonoid (TF) content, total antioxidant capacity (TOAC), and antioxidant capacity equivalent to Trolox (TEAC) are presented in Table 5, while DPPH (2,2-diphenyl-1-picrylhydrazyl) radical scavenging, Ferric Reducing Antioxidant Power (FRAP), Copper Ion Reducing Antioxidant Capacity (CUPRAC), and lipid peroxidation inhibition in the β-carotene/linoleic acid system are outlined in Table 6.

### 2.8. Pearson Correlation Analysis

**Figure 4:**
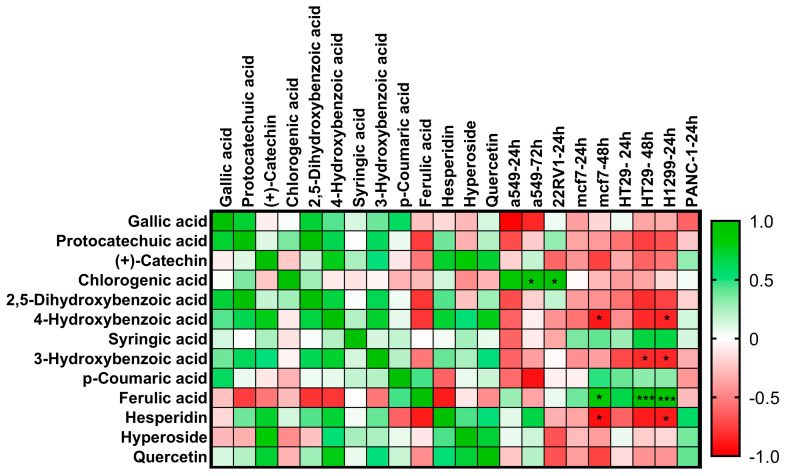
Pearson correlation analysis was employed to create a heat map of the correlation coefficient matrix between the IC_50_ doses of the extracts administered to A549, 22RV1, MCF7, HT29, H1299, and PANC1 and the variations in phytochemical levels in *A. pisidicus* methanol and water extracts. A bidirectional correlation heat map was created. Green and red color labels indicate positive and negative correlations, respectively. Significant values (*, *p* < 0.001; ***, *p* < 0.05) are indicated by those marked “*”; the color label indicates the Pearson correlation coefficient value. The evaluation did not include cell lines for which the IC_50_ dose could not be established.

The most abundant substances in *A. pisidicus* methanol and water extracts compared with other components are hesperidin, 3-hydroxybenzoic acid, and *p*-hydroxybenzoic acid, respectively. The relationship between the variations in compound quantities from the content analysis and the IC_50_ values derived from cell viability analysis was assessed using Pearson analysis. The Pearson correlation indicates that chlorogenic acid (r = 0.9320, *p* = 0.05) exhibited a positive correlation after 72 h of incubation in A549 cells. In H1299 cells, *p*-hydroxybenzoic acid (r = −0.8401, *p* = 0.05), 3-hydroxybenzoic acid (r = −0.8341, *p* = 0.04), and hesperidin (r = −0.99, *p* = 0.05) demonstrated negative correlations. Ferulic acid (r = 0.9840, *p* = 0.001) displayed a positive correlation following 24 h of incubation. At the end of the 24 h incubation period, in HT29 cells, 3-hydroxybenzoic acid exhibited a negative correlation (r = −0.7098, *p* = 0.04), but ferulic acid demonstrated a positive correlation (r = 0.9922, *p* = 0.001). Following a 48-h incubation in MCF7 cells, both *p*-hydroxybenzoic acid (r = −0.8801, *p* = 0.04) and hesperidin (r = −0.8708, *p* = 0.04) exhibited a negative correlation, whereas ferulic acid (r = 0.8564, *p* = 0.05) demonstrated a positive correlation (Figure 4 and Table S1).

### 2.9. Docking Studies

The Autodock Vina (version 1.2.0, The Scripps Research Institute, La Jolla, CA, USA) software was used to calculate the binding energies and poses of the compounds under study in relation to the MDM2 and XIAP proteins. The results are presented in Table 7. Docking studies revealed that chlorogenic acid showed the best binding affinity to XIAP (−6.6 kcal/mol), while hesperidin showed the strongest binding affinity to MDM2 (−7.2 kcal/mol). Furthermore, quercetin, hyperoside, and catechin showed notable binding affinity to MDM2 and XIAP (Table 7).

**Figure 5:**
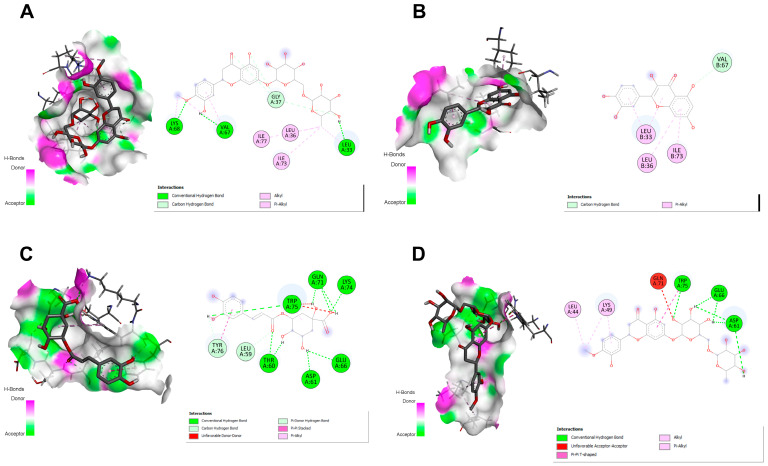
Three-dimensional interactions of (**A**) MDM2-hesperidin; (**B**) MDM2-quercetin; (**C**) XIAP-chlorogenic acid; and (**D**) XIAP-hesperidin complexes.

In addition, the interaction profiles of the two compounds with the highest binding affinity to MDM2 and XIAP, as well as 2D and 3D plots of these interactions, are shown in Figure 5. According to Figure 5A, the interacting residues in the MDM2-hesperidin complex showed a binding energy of −7.2 kcal/mol, so these interactions are defined as a carbon–hydrogen bond with Gly37 and three hydrogen bonds with Lys68, Val67, and Leu33 (Figure 5A). The MDM2-quercetin complex contained a carbon–hydrogen bond (Val67) with a binding energy of −6.9 kcal/mol (Figure 5B).

The XIAP-chlorogenic acid complex showed a low binding energy of −6.7 kcal/mol, with the binding position having two carbon-hydrogen bonds (Leu59, Tyr76) and nine hydrogen bonds (Thr60, Asp61, Glu66, Gln71, Lys74, Trp75) (Figure 5C). With a binding energy of −6.6 kcal/mol, the XIAP-hesperidin complex has six hydrogen bonds (Thr60, Asp61, Glu66, Gln71, Lys74, and Trp75) (Figure 5D). As a result of the redocking experiments, the RMSD values of MDM2 and XIAP proteins were confirmed to be 1.42 Å and 1.26 Å, respectively.

## 3. Discussion

The literature search did not show any previous study on the chemical profile of *A. pisidicus*, and thus, the current work is considered the first investigation of the phytochemistry of this species. Among the 13 phytochemicals identified in the extracts, hesperidin (147–40174 µg/g extract) and hyperoside (363–2677 µg/g extract) were determined to be the main components. Some *Astragalus* species have been reported to contain hesperidin and hyperoside as main constituents. Aydemir et al. (2024), in their phytochemical study on methanol extracts of various parts of *Astragalus gymnolobus* Fisch., similarly found that methanol extracts from roots and stems had higher content of hesperidin and hyperoside compared with other parts [29]. According to Sarikurkcu et al. (2020), in their investigation of the phytochemical composition of *A. gymnolobus*, the methanol extract was found to be abundant in hesperidin, hyperoside, (+)-catechin, vanillic acid, protocatechuic acid, and *p*-hydroxybenzoic acid [30]. Additionally, these two phytochemicals have been reported as main constituents in other *Astragalus* species [31,32,33]. It can be said that the results obtained in this study exhibit a significant degree of similarity with the literature data.

Among all tested parts of *A. pisidicus*, the highest phenolic content was detected in the Lw extract as 364.42 ± 10.37 mg GAE/g, whereas the highest flavonoid content was detected in the Lm extract as 70.14 ± 4.18 mg QE/g. The outcomes of the antioxidant activity assessments seem to corroborate this situation. TOAC and TEAC of the leaf extracts, both methanol and water, were generally found to be higher than the other extracts. The concentration of antioxidants and their synergistic interactions with other phytochemicals can affect the variations in the antioxidant activity of plant extracts [34]. The increase in total phenolic and flavonoid content may correlate with an enhancement in antioxidant capabilities. The reducing capacities of the extracts were assessed using the CUPRAC and FRAP test systems. Lm extract exhibited the highest activity in both tests. Moreover, the ferric reducing antioxidant power of Lm extract of *A. pisidicus* (4.19 ± 0.08 mM FeSO_4_/g) was higher than that of *A. gymnolobus* Lm extract (3.72 ± 0.09 mM FeSO_4_/g) used in our previous study. However, the copper ion-reducing effect of *A. gymnolobus* Lm extract (148 ± 4.63 mg AAE/g) was higher than that of *A. pisidicus* Lm extract (140 ± 7.36 mg AAE/g) [33]. Inhibiting lipid peroxidation effect in β-carotene/linoleic acid system (% activity 1 mg/mL) of Lw and Lm was calculated as 91.37 ± 1.73 and 88.47 ± 2.62, respectively. DPPH, a stable nitrogen-centered and lipophilic free radical, has been extensively utilized as a reagent for assessing the free radical scavenging capabilities of compounds [35]. The extracts can be ranked from the highest to the lowest DPPH scavenging capabilities (EC_50_ µg/mL) as 768 ± 4.3 (Fw) and 119 ± 10.11 (Lm), respectively.

This study presents, for the first time, data on the cytotoxic effects of various parts of *A. pisidicus* methanol and water extracts against different cancer cell lines. The IC_50_ values indicate the extract concentration at which 50% of the cells’ growth is inhibited. The National Cancer Institute (NCI) classifies a crude extract based on its cytotoxicity as active, moderately active, or inactive, with IC_50_ values categorized as follows: less than 20 μg/mL indicates activity, between 2 and 100 μg/mL indicates moderate activity, and values exceeding 100 μg/mL indicate inactivity [31]. Our findings unequivocally indicate that various extracts display cytotoxic effects across distinct cell types. Considering the aforementioned conditions of NCI, we evaluated Fm (IC_50_ = 9.57 µg/mL), Rm (IC_50_ = 14.89 µg/mL), and Sm (IC_50_ = 9.57 µg/mL) as active crude extracts on H1299, HT-29, and PANC-1 cells, while Rm (IC_50_ = 32.057 µg/mL) and Fm (IC_50_ = 64.25 µg/mL) were assessed as moderately active on MCF-7 and 22RV1 cells, respectively, among all the extracts. Unfortunately, the extracts did not exhibit cytotoxic effects on HeLa and MDAMB-231 cells. The ability of any evaluated substance to effectively and selectively limit cancer cell growth and/or proliferation while avoiding damaging effects on normal cells enhances its anti-cancer efficacy. The ability of any evaluated substance to effectively and selectively limit cancer cell growth and/or proliferation while avoiding damaging effects on normal cells enhances its anti-cancer efficacy. The sole assessment of the cytotoxic activity of the examined extracts exclusively in cancer cells is inadequate to assert that the extract possesses antiproliferative or anticancer properties. The extracts, which we classified as active or moderate according to NCI, demonstrated cytotoxic effects exclusively in cancer cells, with no cytotoxic activity observed in 293T cells, which represent non-cancerous cells. Consequently, it can be asserted that they exhibit selective cytotoxicity. According to the selective cytotoxicity findings, *A. pisidicus* extracts exhibited anticancer activity against cancer cells in a dose-dependent manner, indicating the plant’s considerable potential in anti-cancer drug development.

Numerous studies demonstrate that *Astragalus* species possess cytotoxic and antiproliferative effects on cancer cells [36]. Various extracts derived from distinct sections of *Astragalus* species and isolated compounds exhibited cytotoxic or antiproliferative effects on cancer cells by influencing diverse signaling pathways [36,37,38,39]. According to NCI criteria, extracts obtained from *A. pisidicus* were inactive on the non-small cell lung cancer (NSCLC) line A549 (wild type *p53* gene). However, Fm was evaluated as an active crude extract in NSCLC H1299 (*p53* null) [40]. Consequently, we propose that the active Fm extract may not have induced its cytotoxic effects via the p53 protein and associated pathways. Conversely, in breast cancer cells, none of the extracts exhibited any effect on MDAMB231 cells expressing high levels of a mutant p53 protein, while Rm was identified as the active extract in MCF-7 cells (wild type *p53* gene) [40]. The findings in breast cancer cells indicate that p53 and associated proteins may play a role in the mechanism of action. Based on this information, we examined the binding energies of the most abundant substances in the extracts to the MDM2 and XIAP proteins, which are associated with p53, through docking studies. MDM2 and XIAP play a crucial role in the regulation of apoptosis. According to certain research, hesperidin and chlorogenic acid induce apoptosis by downregulating the proteins Bcl-2 and XIAP [41,42]. Additionally, hesperidin inhibited the interaction between p53 and MDMX, increased p53 expression, and induced apoptosis in A549 and NCI-H460 cells [43]. As reported by Ghorbani et al. (2012), hesperidin promotes p53 accumulation and inhibits Bcl-2 and XIAP expression [44]. Molecular docking is a crucial approach in the study of protein–ligand interactions and the discovery and development of new drugs. A docking study was conducted using Autodock Vina to evaluate the binding capacity of the compounds in *A. pisidicus* extract to the anticancer target proteins XIAP and MDM2. Hesperidin (−7.2 kcal/mol) and quercetin (−6.9 kcal/mol) showed the lowest binding affinity to MDM2. In our previous study, we examined the molecular interactions of compounds in *A. gymnolobus* extract with proteins BCL-2, CDK1, HDAC2, TNFα, and PBP2a, where hesperidin exhibited the highest binding affinity to the target proteins [29]. The MDM2-hesperidin complex contains one carbon–hydrogen bond, three hydrogen bonds, and three hydrophobic interactions. One carbon–hydrogen bond and four hydrophobic interactions were present in the MDM2-quercetin complex. The findings of Verma et al. (2013) indicate that quercetin forms stable hydrophobic interactions with MDM2, leading to the dissociation of p53 from the complex [45]. The lowest binding affinity to XIAP was observed for chlorogenic acid (−6.7 kcal/mol) and hesperidin (−6.6 kcal/mol), which exhibited two carbon–hydrogen bonds and nine hydrogen bonds in the XIAP-chlorogenic acid complex and six hydrogen bonds in the XIAP–hesperidin complex.

A statistically significant correlation exists between the variations in the concentrations of major compounds in the methanol and water extracts of *A. pisidicus* and the IC_50_ values obtained from A549, HT29, H1299, MCF7, and 22RV1 cell lines. Ferulic acid exhibits a strong positive correlation with cytotoxicity after 24 h of incubation in H1299 cells and 48 h in HT29 cells. Research examining the cytotoxic effects of ferulic acid on cancer cells is documented in the literature, demonstrating a strong positive correlation [46,47,48,49]. Ferulic acid has demonstrated multiple biological actions, particularly in relation to oxidative stress, inflammation, vascular endothelial injury, fibrosis, apoptosis, and platelet aggregation [50]. A study on ferulic acid in colon cancer indicated that exposure resulted in dose-dependent cytotoxicity and induced apoptosis in HT29 cells [48]. Bakholdina et al. (2019) demonstrated that ferulic acid derivatives induced cytotoxic effects on HCT116 colon cancer cells [47]. Ferulic acid has also been demonstrated to enhance radiation sensitivity in A549 lung cancer cells and to elevate the expression of pro-apoptotic proteins BAX and p53, in addition to inducing cell cycle arrest [51]. Ferulic acid stimulates the production of cell cycle-associated proteins, including p53 and p21, and initiates apoptosis. The possible apoptotic impact of ferulic acid is attributed to modified expression of caspase-3, -8, -9, BCL2, and BAX [52,53].

Chlorogenic acid (CGA) is a natural polyphenol consisting of caffeic acid and quinic acid. CGA is a principal active component present in coffee, fruits, and vegetables and is extracted from traditional Chinese medicine. CGA has demonstrated cytotoxic or inhibitory effects against various cancer types, including hepatocarcinoma, breast cancer, brain cancer, leukemia, skin cancer, and particularly lung cancer [54]. The study by Yamagata et al. (2018) found that 30 and 50 μM chlorogenic acid inhibited A549 cell proliferation by 27% and 39%, respectively, and reduced BCL2 expression in A549 cells. *casp3* gene expression was dramatically elevated in A549 cells following chlorogenic acid therapy [55]. Research on ferulic acid and chlorogenic acid in the literature corroborates our Pearson correlation analysis findings.

Apoptosis is a form of programmed cell death crucial for preserving tissue homeostasis through the removal of unnecessary, excess, and damaged cells. The dysregulation of apoptosis contributes to the pathogenesis of various diseases, including neurodegenerative disorders marked by excessive apoptosis and cancer, which is characterized by the accumulation of cells that inadequately activate the apoptotic machinery and evade apoptosis. Cancer cells evade apoptosis by impairing the mechanisms of cell death, resulting in a significant survival advantage [56,57,58]. Consequently, triggering apoptosis in neoplastic cells is a major mechanism underlying the activity of any tested extract and/or compound. Apoptosis is mediated by two principal pathways: the intrinsic pathway and the extrinsic pathway. Both pathways culminate in the activation of cysteine aspartyl-specific proteases, known as ’caspases’ [57]. As a response to diverse apoptotic triggers, ’initiator’ caspases (caspase-2, -8, -9, or -10) become activated. Initiator caspases cleave and activate the zymogenic forms of executioner caspases (e.g., caspase-3 or -7), leading to the proteolytic cleavage of certain cellular substrates and, ultimately, cell death. The cleavage (activation) of executioner caspases is a defining characteristic of apoptosis. Apoptosis occurs via two pathways: extrinsic and intrinsic. The extrinsic pathway involves the activation of initiator caspases-8 and -10 via the assembly of a death-inducing signalling complex (DISC) upon the interaction of extracellular ligands, such as Fas and tumor necrosis factor (TNF), with cell surface receptors. The intrinsic pathway of apoptosis involves mitochondrial outer membrane permeabilization (MOMP), which initiates the release of proapoptotic proteins, such as cytochrome c and second mitochondria-derived activator of caspases (SMAC), from the mitochondrial intermembrane space into the cytoplasm. In the cytoplasm, cytochrome c associates with the adaptor protein apoptotic protease-activating factor 1 (APAF-1) to create a caspase-9-activating complex known as the apoptosome. SMAC enhances caspase activation triggered by cytochrome c by attaching to and neutralizing the X-linked inhibitor of apoptosis protein (XIAP), which inhibits caspase-3, -7, and -9. Cancer cells experience persistent stress, such as oncogenic stress, genomic instability, and cellular hypoxia, in contrast to normal cells. In reaction to apoptotic stimuli, the intrinsic pathway of apoptosis is typically activated. Cancer cells frequently evade this cellular response by inactivating the apoptotic pathways. Consequently, it is strongly indicated that the prevention of apoptosis is pivotal in the survival of cancer cells and the progression of tumors [58]. The detection of apoptosis serves as a crucial signal in evaluating prospective new pharmaceuticals and the overall cytotoxicity of substances [59]. Moreover, triggering apoptosis in cancer cells represents a significant strategy for cancer therapies. Herein, we assessed whether the cytotoxic extracts, with established IC_50_ values, induced apoptosis in cancer cells by analyzing the activity changes in caspase-2, -3, -6, -8, and -9 enzymes in comparison to the control group. The results of this study demonstrate that the various extracts tested in different cancer cell types significantly enhance the activities of caspases linked to either the intrinsic or extrinsic apoptosis pathways. In this regard, it is evident that various extracts of *A. pisidicus* induce apoptotic processes in distinct cancer cells.

DNA fragmentation is the main feature of apoptosis and is consequently utilized as an indicator of this process. Double-stranded DNA is broken by the DNA fragmentation factor (DFF), a heterodimer composed of a 40 kDa catalytic component (DFF40) and a 45 kDa regulatory subunit (DFF45). DFF40 exhibits endonuclease activity at neutral pH in the presence of Mg^2+^ and specifically cleaves double-stranded DNA, showing a preference for A/T-rich regions. The inhibitor DFF45, which also serves as a chaperone for DFF40 during its production under normal conditions, blocks DFF40. During apoptosis, procaspase 3 is cleaved into active caspase 3, which then cleaves the DFF45–DFF40 complex, activating DFF40 to cleave nuclear DNA into internucleosomal fragments of 180 bp in size and its multiples. DNA fragmentation serves as a definitive marker of apoptosis; hence, techniques have been employed to assess DNA fragmentation in cells to unequivocally demonstrate that the mode of cell death is apoptosis [59,60,61,62,63]. Our results demonstrate that the extracts, which significantly elevate caspase activity, also enhance DNA fragmentation, so indicating that the extracts induce apoptosis in cells.

Antimicrobial activities of methanol and water extracts obtained from different parts of *A. pisidicus* were evaluated against 16 bacterial strains. No other study was found in the literature investigating the antimicrobial activity of *A. pisidicus*. In this respect, the antimicrobial activity results we obtained as a result of this study are the first. Methanol extracts obtained from different parts of the plant exhibited varying activities against gram-positive bacteria. However, none of the extracts obtained using water were effective against the tested bacteria. Similarly, Jaradat et al. (2017) reported that methanol extractions of *Astragalus pelecinus* (L.) Barneby were more effective than aqueous extractions [64].

In our study, it was determined that methanol extract was effective only against gram-positive bacteria. Shahrivari-Baviloliaei et al. (2024) also reported that the extracts from *A*. *membranaceus* Fisch. ex Bunge were only effective against gram-positive bacteria, *S. aureus* [65]. Unlike these results, different *Astragalus* species were found to be effective against gram-negative bacteria such as *Escherichia coli*, *Pseudomonas aeruginosa*, and *Salmonella typhimurium* [66,67,68].

An inhibitory activity of methanol extracts obtained from different parts of *A. pisidicus* was observed in the 8–12 mm diameter range against all gram positives except *S. epidermidis*. Among these bacteria, the highest sensitivity was observed in *S. pyogenes*. Similar results were obtained in our previous study in which we studied the antimicrobial activity of *A. gymnolobus* [29]. In parallel with these results, *Astragalus brachypterus* [27] and *Astragalus gymnalopecias* [69] extracts were found to be effective only against *S. pyogenes*.

When methanol extracts obtained from different parts of the plant were compared, it was determined that WPm extract showed the highest antimicrobial activity. It is known that the inhibitory effects of plant extracts depend on compounds such as phenolics, flavonoids, terpenoids, alkaloids, saponins, and glycosides [70]. In this study, the higher amounts of phenolic compounds and flavonoids in the whole plant were associated with the broad spectrum of antimicrobial activity observed in WPm extract compared with other methanol extracts.

## 4. Materials and Methods

### 4.1. Plant Material

*A. pisidicus* was collected from Korkuteli to Fethiye road, Kızılaliler village, fieldside, Antalya province in Turkey during the flowering period in May 2014, 36°59′03.7″ N, 29°57′50.5″ E. The taxonomic identification of the species was carried out by Dr. R. Süleyman Göktürk (Faculty of Science, Akdeniz University, Antalya, Turkey), where a voucher specimen has been deposited (Herbarium number: 7701).

### 4.2. Extraction

The shade-dried whole plant, along with its separated flowers, roots, stems, and leaves, was pulverized to pass through a 0.4 mm sieve using a dry grinder to achieve a uniform particle size. A total of 50 g of each ground part, along with the whole plant, were individually extracted with both water (100 mL) and methanol (100 mL). The extracts were filtered through Whatman No. 1 paper in a Buchner funnel. The resulting extracts were concentrated utilizing a rotary evaporator (Rotavator, T < 40 °C) and lyophilized subsequently as described previously (Virtis 2 K, T = −60) [71] to yield powder form extracts. The extracts were stored at −20 °C until further processing. Immediately before the in vitro assays, the extracts were resuspended in PBS and centrifuged at 2000× *g* rpm to discard any insoluble material. Samples were sterile-filtered through 0.22 µm filters.

### 4.3. Identification of Phenolic Compounds by LC–ESI–MS/MS

The phytochemical compositions of the extracts were analyzed quantitatively. Quantitative analyses were carried out by using LC–ESI–MS/MS technique, which was previously developed and validated by Cittan and Çelik (2018) [72]. A simple, rapid, reproducible, and sensitive method, which was previously developed and validated, was used for the simultaneous determination of 30 phenolic compounds using LC–ESI–MS/MS. An Agilent Technologies (Santa Clara, CA, USA) 1260 Infinity liquid chromatography system hyphenated to a 6420 Triple Quad mass spectrometer was used for quantitative analyses. Chromatographic separation was carried out on a Poroshell 120 EC-C18 (100 mm × 4.6 mm I.D., 2.7 μm) column. The mobile phase configuration (0.1% formic acid/methanol) was selected on the basis of the better chromatographic resolution of isomeric compounds. On the other hand, the selected mobile phase configuration also provided higher sensitivity for many of the phenolic compounds. As a result, the mobile phase was made up of solvent A (0.1%, *v*/*v* formic acid solution) and solvent B (methanol). The gradient profile was set as follows: 0.00 min 2% B eluent, 3.00 min 2% B eluent, 6.00 min 25% B eluent, 10.00 min 50% B eluent, 14.00 min 95% B eluent, 17.00 min 95% B, and 17.50 min 2% B eluent. The column temperature was maintained at 25 °C. The flow rate was 0.4 mL min^−1^, and the injection volume was 2.0 μL. The tandem mass spectrometer was interfaced to the LC system via an ESI source. The electrospray source of the MS was operated in negative and positive multiple reaction monitoring (MRM) mode, and the interface conditions were as follows: capillary voltage of −3.5 kV, gas temperature of 300 °C, and gas flow of 11 L min^−1^. The nebulizer pressure was 40 psi.

In negative and positive multiple reaction monitoring (MRM) mode, the peaks of the analytes were identified by comparing the retention time, together with the monitoring ion pairs, in an authentic standard solution. LC-ESI–MS/MS parameters and calibration curves of the method were presented in Table 8 and Table 9.

### 4.4. Cell Culture

MDAMB-231 (ATCC^®^ HTB-26™, estrogen receptor negative human breast cancer cell line), MCF-7 (ATCC^®^ HTB-22, estrogen receptor positive human breast cancer cell line), 22RV1 (ATCC^®^ CRL-2505, human epithelial prostate carcinoma cell line), A549 (ATCC^®^ CRM-CCL-185, human epithelial lung carcinoma cell line), H1299 (ATCC^®^ CRL-5803, human NSCLC cell line), PANC-1 (ATCC^®^ CRL-1469, human pancreatic epithelioid carcinoma cell line), HT-29 (ATCC^®^ HTB-38, human colorectal adenocarcinoma cell line), HeLa (ATCC^®^ CRM-CCL-2, human epithelial cervix adenocarcinoma cell line) and 293T (ATCC^®^ CRL-1573, human embryonic kidney cell line) were purchased from the Ameri-can Type Culture Collection (ATCC) (Rockville, MD, USA). The cells were cultured in RPMI 1640 media enriched with 10% fetal bovine serum (FBS), 2 mM L-glutamine, 1 mM sodium pyruvate, and 0.02 mM non-essential amino acids. The cells were maintained at 37 °C in a humidified environment with 5% CO_2_. The cells were preserved in a deep freezer at −80 °C in a solution composed of 95% medium and 5% DMSO.

### 4.5. Cell Proliferation (WST-1) Assay

Cell proliferation was assessed according to the manufacturer’s protocol using the WST-1 cell proliferation kit (Roche, Basel, Switzerland, Kat. No: 11 644 807 001). This is a colorimetric assay that is based on the cleavage of a tetrazolium salt, MTS, by mitochondrial dehydrogenases to form formazan in viable cells [73]. Cells were seeded in 96-well plates at a final concentration of 1 × 10^4^ cells/well in 100 μL of complete culture medium and allowed to attach for 24 h. Once the cells reached 80–90% confluence, the media was removed, and the cells were treated with extracts prepared in 1% FBS-supplemented complete medium and incubated at 37 °C for 24, 48, and 72 h. Cell viability in the first single column was determined and recorded as time zero (T_0_) immediately following the treatment. The medium containing 1% FBS was used as the negative control, and each treatment was conducted in eight replicates. At the end of the incubation, the medium was gently aspirated to terminate the experiment; a total volume of 10 μL (5 mg/mL) of CCK-8 solution was added to each well and further incubated under the same conditions for 4 h. The absorbances at 450 nm were measured in a microplate reader (Thermo Labsystem, Waltham, MA, USA), using wells without cells as background. The sample readings were calculated by subtracting the average of background absorbances. The half-maximal inhibitory concentration (IC_50_) of each extract was derived by a nonlinear regression model (curve fit) based on a sigmoidal-dose response curve (variable slope) and computed using Graph-Pad Prism, version 4.00 (Graph-Pad Software, San Diego, CA, USA). Cell viability was calculated using the following formula: Cell viability (%) = (OD_treatment_ − OD_blank_)/(OD_control_ − OD_blank_) × 100% [74].

### 4.6. DNA Fragmentation

The quantification of cytoplasmic histone-associated DNA fragments (mono- and ol-igonucleosomes) following the induction of cell death by extracts was conducted using the Cellular DNAFragmentation ELISA kit (Roche Diagnostics, Rotkreuz, Switzerland, 11 585 045 001) as per the manufacturer’s instructions. Briefly, 5 × 10^5^ cells/mL were labeled with 10 µM BrdU and incubated for 4 h at 37 °C. The cells were centrifuged at 750× *g* for 10 min. The supernatant containing BrdU was discarded, and the cells were resuspended in BrdU-free RPMI. A total of 100 µL of BrdU-labeled cells (1 × 10^5^ cells/mL) were seeded in flat-bottom microtiter plates (MTP) and treated with extracts (¼ IC_50_, ½ IC_50_, IC_50_, 2× IC_50_, 4× IC_50_) for 6 h. Following the incubation period, the supernatant was discarded, and 200 µL of 1× incubation solution was added to each well. The plates were incubated at room temperature for 30 min to lyse the cells and thereafter were centrifuged at 1000× *g* for 10 min. The supernatants were transferred to new eppendorf tubes to isolate the DNA fragments from the cytoplasm. According to the ELISA protocol, 100 µL of the supernatant was transferred to anti-DNA-coated flat-bottom 96-well microtiter plates and incubated for 90 min at room temperature, after which DNA was denatured using nuclease treatment. A total of 100 µL of anti-BrdU-POD conjugate solution was applied per well, and the samples were further incubated for 90 min. The plates were washed five times with 1X wash buffer. Substrate (TMB, 100 µL) solution was added for blue color development. After the addition of 25 µL of stop solution, the absorbances at 450 nm were measured in a microplate reader (Thermo Labsystem, USA) [75].

### 4.7. Caspase Assay

Caspase activity was determined using the ApoTarget caspase colorimetric protease assay sampler kit (Invitrogen Corporation, KHZ 1001, Waltham, MA, USA) according to the manufacturer’s instructions. Briefly, cells were treated with extracts (¼ IC_50_, ½ IC_50_, IC_50_, 2× IC_50_, 4× IC_50_) for 24 h. The cells were subsequently harvested, rinsed in PBS, and lysed in 50 μL of lysis buffer on ice for 10 min. After centrifugation, the supernatant containing 150 μg of proteins was incubated with 200 μM of caspase-2 (Ac-VDVAD-pNA), caspase-3 (Ac-DEVD-pNA), caspase-6 (Ac-VEID-pNA), caspase-8 (Ac-IETD-pNA), and caspase-9 (Ac-LEHD-pNA) substrates, respectively, in reaction buffer at 37 °C for 1 h in a 96-well flat-bottom plate. Levels of released pNA were then measured at 405 nm wavelength with ELISA plate reader (Thermo Labsystem, USA). The fold increase in caspase-2,-3,-6, -8, and -9 activities was determined by direct comparison to the level of untreated control [76].

### 4.8. Antimicrobial Activity

#### 4.8.1. Bacterial Strains

The antimicrobial activity of the extracts was evaluated against a variety of clinically relevant American Type Culture Collection (ATCC) strains. The reference strains were as follows: *Staphylococcus aureus* ATCC 25923, *Staphylococcus aureus* ATCC 29213, *Staphylococcus aureus* ATCC 43300, *Staphylococcus epidermidis* ATCC 12228, *Enterococcus faecalis* ATCC 29212, *Enterococcus faecalis* ATCC 51299, *Streptococcus pyogenes* ATCC 19615, *Escherichia coli* ATCC 25922, *Escherichia coli* ATCC 35218, *Klebsiella pneumoniae* ATCC 13883, *Klebsiella pneumoniae* ATCC 700603, *Enterobacter cloacae* ATCC 23355, *Serratia marcescens* ATCC 8100, *Proteus vulgaris* ATCC 13315, *Salmonella typhimurium* ATCC 14028, *Pseudomonas aeruginosa* ATCC 27853. The bacterial strains were prepared for testing by culturing overnight at 37 °C on Blood Agar (Becton Dickinson, Franklin Lakes, NJ, USA).

#### 4.8.2. Disc Diffusion Method

The antibacterial activity of the extracts was evaluated using the CLSI (Clinical and Laboratory Standards Institute) standard disc diffusion method [77]. The bacterial suspensions were prepared in accordance with the 0.5 McFarland standard (Bio-Merieux, Marcy l’Etoile, France), with a standard density of 1.0 × 10^8^ cells per mL in a 0.9% NaCl solution. Thereafter, the prepared bacterial suspensions were spread on Mueller–Hinton agar (Merck KGaA, Darmstadt, Germany) using sterile swabs. A quantity of 20 µL of the sterilized extracts, which were filtered through a 0.22 µm membrane, was impregnated onto sterile paper discs of diameter 6 mm. The standard antibiotic discs recommended by the CLSI were used as a positive control, while a blank disc was utilized for sterility controls, and methanol without the extract was employed as a negative control. Both samples were placed on the same plates, which were then incubated at 37 °C for 24 h. Thereafter, the inhibition zones were measured in millimeters. Each experiment was set up in quadruplicate.

#### 4.8.3. Broth Microdilution Method

In order to determine the minimum inhibitory concentration (MIC) values of the extracts, a broth microdilution method was employed [78]. Extracts were dissolved in Mueller Hinton Broth (MHB, Merck KGaA) at a concentration of 16.384 μg/mL. Subsequently, a series of serial dilutions of the extract were prepared in a 96-well microtiter plate, with concentrations ranging from 1 to 8192 μg/mL. The suspension of bacterial strains in cation-adjusted MHB was prepared at a final density of 5 × 10⁵ cfu/mL, and the selected bacterial strain was inoculated into each well. Each microdilution plate was prepared to comprise a growth control (MHB+bacteria) and a sterility control (MHB only). The microdilution plates were incubated at 35 ± 2 °C for 18–24 h. The MIC values were obtained by comparing the bacterial growth density in the wells containing antibiotics with that in the control wells used in each test set. Each experiment was set up in quadruplicate.

### 4.9. Antioxidant Activity

#### 4.9.1. Total Phenolic Contents of the Extracts

The total phenolic content of *Astragalus* extracts was assessed using the Folin–Ciocalteu reagent, following the Singleton and Rossi technique, with gallic acid as the standard [79]. The Folin–Ciocalteu reagent was employed to create a blue molybdenum–tungsten complex with the phenolic compounds present in the extracts. Total phenol concentration was quantified as micrograms of gallic acid equivalents per milligram of the extracts.

#### 4.9.2. Determination of Total Flavonoid Contents

The total flavonoid concentration was quantified using the aluminum chloride colorimetric method [65]. The total flavonoid concentration was quantified as quercetin equivalents (mg RE/g).

#### 4.9.3. DPPH Radical Scavenging Assay

The free radical scavenging activity of *Astragalus* extracts was assessed using the DPPH technique. DPPH is a stable free radical utilized to assess the free radical scavenging capacity of natural substances. DPPH is converted to yellow diphenyl picryl hydrazine when a hydrogen-donating antioxidant molecule is present. The absorbance of the resultant yellow chemical was quantified colorimetrically [66]. The percentage inhibition was calculated as follows: percentage inhibition = [(A0 − A1)/A0] × 100, where A0 is the absorbance of the blank sample and A1 is the absorbance of the sample.

#### 4.9.4. Determination of CUPRAC

The extracts’ ability to reduce copper ions was assessed through a method that involves the conversion of Cu^+2^ to Cu^+1^ in the presence of neocuproin, followed by complexation with neocuproin [80].

#### 4.9.5. Ferric Reducing Antioxidant Power (FRAP)

The reduction in the Fe^+3^ ion is frequently employed to assess the electron-donating capacity and various antioxidant properties. The iron-reducing capacity of the extracts was assessed using a method that detects the dark blue color produced when the ferric 2,4,6-tripridyl-s-triazine complex (Fe^+3^-TPTZ) is reduced to its ferrous form (Fe^+2^-TPTZ) [81].

#### 4.9.6. Determination of Lipid Peroxidation Inhibitory Effect in β-Carotene/Linoleic Acid System

This technique relies on the decolorization of β-carotene caused by radicals generated from linoleic acid in an emulsion. The presence of any antioxidant inhibits discoloration, and spectrophotometric analysis can quantify the reaction [82].

#### 4.9.7. Determination of Total Antioxidant Capacity

This method involves the conversion of Mo^+6^ to Mo^+5^, leading to the creation of a green phosphate/Mo^+5^ complex [83].

#### 4.9.8. Determination of Antioxidant Capacity Equivalent to Trolox

The ABTS radical is a cationic free radical generated from the reaction of 2,2′-azino-bis(3-ethylbenzothiazoline-6-sulphonic acid) (ABTS) and potassium persulfate. The intense green color of the ABTS radical diminishes in the presence of a hydrogen-donating antioxidant. Results are quantified as equivalent to Trolox in aqueous solution [84].

### 4.10. Pearson Correlation Analysis

The relationship between the changes in the amounts of phytochemicals in the extracts of *A. pisidicus* and the applied IC_50_ doses of the extracts was examined by Pearson correlation analysis using Graphpad Prism 9 software. The heat map was prepared bidirectionally in order to observe the variability between the changes in the amounts of phytochemicals and the IC_50_ doses [85,86].

### 4.11. Molecular Docking

The mouse double minute 2 (MDM2) is acknowledged for its pivotal role in regulating cellular growth, apoptosis, DNA repair, and metastasis in cancer cells. The inhibition of MDM2 stabilizes p53, thereby activating p53 target genes and resulting in cell cycle arrest and apoptosis [87]. X-linked inhibitors of apoptosis proteins (XIAP) specifically inhibit the activity of caspases 3, 7, and 9, which are responsible for inducing the mitochondrial apoptotic pathway [88]. Consequently, MDM2 and XIAP were identified as potential targets for docking studies.

The crystal structures of MDM2 (PDB ID: 4ERE) and XIAP (PDB ID: 5OQW) were obtained from the Protein Data Bank (PDB). The preparation of protein structures was conducted using UCSF Chimera (version 1.16, UCSF, San Francisco, CA, USA) for the docking process. Following the removal of the native ligand and water, the 3D structure was completed with the addition of polar hydrogen atoms and AMBER ff14SB partial charges. The structures of the compounds were obtained from PubChem (Table 7), and the compounds were then minimized in energy via the UFF (Universal Force Field) method, which was performed using Avogadro (version 1.2.0). Subsequently, the ligand preparation was completed using UCSF Chimera. A grid box was generated in accordance with the binding site of the co-crystallized ligand. The potential binding modes between the protein and the ligand were calculated based on the grid box. Subsequently, molecular docking was performed utilizing AutoDock Vina (version 1.2.0, The Scripps Research Institute, La Jolla, CA, USA) software. The visualization of the results and analysis of protein-ligand interactions were conducted with BIOVIA Discovery Studio Visualizer (version 21.1.0.20298, Dassault Systèmes, Vélizy-Villacoublay, France).

The co-crystallized ligands were extracted and re-docked to validate the docking process. Their quality was evaluated by calculating their root-mean-square deviation (RMSD) values using the DockRMSD webserver [89].

### 4.12. Statistical Analysis

The values obtained after each application and each set were checked for compliance with the normal distribution and variation homogeneity. Parametric and non-parametric test methods were used for the appropriate and non-parametric tests, respectively. ANOVA-MANOVA was used in the analysis of the data that meet the parametric hypothesis criteria; otherwise, Kruskal–Wallis hypothesis tests were used. In the tables to be prepared for each analysis, the probability of statistical significance (reliability level) (*p*), mathematical degree of freedom (df), and calculated test values are given. The statistical error (α) was taken as 0.05 for each test. The results were interpreted separately, considering their significance. Hypothesis tests were performed using SPSS for Windows 17.0.0–19.00 SPSS Inc. 1989–2010 and Microsoft Excel XP. Prepared datasets were recorded on stationary media for later use. Depending on the results obtained from the experiments in this study, the above-mentioned package programs were used during the calculation of the dose-dependent IC_50_ value. In probit-based statistical evaluations, the concentrations that inhibited viability between 25% and 75% in the trials were pre-evaluated. The results of the trials in which the doses capable of providing 50% inhibition can be calculated from these preliminary evaluation results were used in the tables, graphs, and evaluations; others were not considered. In the data obtained from the statistical program, Instat 3, used for drawing the graphs; *: *p* < 0.05, **: *p* < 0.01, and ***: *p* < 0.001 were used to show the importance. Quantitative analyses using the LC–ESI–MS/MS technique were carried out in triplicate. In order to determine the degree of statistical difference, Tukey’s test was used.

## 5. Conclusions

The phytochemical properties, antioxidant, antibacterial, and cytotoxic activities of water and methanol extracts of *A. pisidicus* have been evaluated in vitro for the first time. This study concluded that *A. pisidicus* has important antioxidant properties and induced apoptotic cell death in cancer cells by activating caspases and DNA fragmentation as well. Moreover, these effects were not pronounced in the 293-T, noncancerous cells, indicating clear selectivity towards cancer cell lines. Apart from phenolic compounds, identification of other phytochemical compounds was not carried out in this study. Further investigations are required to determine other constituents of this plant and to elucidate the comprehensive mechanisms underlying the cytotoxic effects.

## Figures and Tables

**Table 1:** The yields of (%) of different parts of *A. pisidicus* methanolic and water extracts.

*A. pisidicus*	Methanol (%)	Water (%)
Root	16.94	18.56
Stem	13.08	9.37
Flower	8.34	7.62
Leaf	20.39	19.89
Whole plant	21.48	16.27

**Table 2:** Concentrations (µg/g extract) of phytochemicals in the methanol and water extracts from various parts of *A. pisidicus*.

	Methanol Extract					Water Extract				
Name	Fm	Sm	Rm	Lm	WPm	Fw	Sw	Rw	Lw	WPw
Gallic acid	20.2 ± 0.3 *^a,x^*	18.6 ± 0.2 *^a,x^*	6.62 ± 0.46 *^c,x^*	12.0 ± 0.7 *^b,x^*	19.7 ± 0.1 *^a,x^*	20.3 ± 0.2 *^a^^,^^x^*	15.8 ± 0.3 *^b,y^*	9.64 ± 0.03 *^e,y^*	11.1 ± 0.3 *^d,x^*	12.5 ± 0.3 *^c,y^*
Protocatechuic acid	192 ± 3 *^a^^,^^x^*	146 ± 1 *^b,x^*	87.4 ± 1.5 *^dx,^*	20.1 ± 0.1 *^e,x^*	111 ± 2 *^c,x^*	214 ± 5 *^a,y^*	139 ± 1 *^b,y^*	85.7 ± 0.2 *^d,x^*	18.9 ± 0.3 *^e,y^*	116 ± 1 *^c,x^*
Gentisic acid	193 ± 3 *^a,x^*	146 ± 3 *^b,x^*	89.9 ± 1.9 *^d,x^*	20.3 ± 0.2 *^e,x^*	113 ± 1 *^c,x^*	190 ± 3 *^a,x^*	141 ± 4 *^b^^,^^x^*	87.2 ± 0.2 *^d,x^*	18.8 ± 0.3 *^e,y^*	120 ± 1 *^c,y^*
(+)-Catechin	8.22 ± 0.08 *^c,x^*	275 ± 4 *^a,x^*	151 ± 4 *^b,x^*	9.11 ± 0.17 *^c^*	141 ± 1 *^b,x^*	2.81 ± 0.12 *^d,y^*	174 ± 6 *^b,y^*	151 ± 2 *^c,x^*	nd	188 ± 4 *^a,y^*
Chlorogenic acid	2.99 ± 0.07 *^e^^,^^x^*	5.48 ± 0.09 *^d,x^*	13.5 ± 0.1 *^a,x^*	6.73 ± 0.17 *^b,x^*	6.12 ± 0.20 *^c,x^*	28.3 ± 0.3 *^a,y^*	5.42 ± 0.44 *^c,x^*	11.5 ± 0.1 *^b,y^*	6.45 ± 0.33 *^c,x^*	10.6 ± 0.3 *^b,y^*
3-Hydroxybenzoic acid	367 ± 4 *^c,x^*	591 ± 3 *^a,x^*	329 ± 3 *^d,x^*	55.7 ± 0.1 *^e,x^*	450 ± 1 *^b,x^*	322 ± 1 *^c,y^*	600 ± 11 *^a,x^*	312 ± 3 *^c,y^*	52.3 ± 1.0 *^d,y^*	458 ± 3 *^b,x^*
*p*-Hydroxybenzoic acid	359 ± 9 *^c,x^*	599 ± 10 *^a,x^*	331 ± 1 *^d,x^*	54.7 ± 1.5 *^e,x^*	451 ± 1 *^b,x^*	326 ± 5 *^c,y^*	589 ± 8 *^a,x^*	310 ± 1 *^c,y^*	50.6 ± 0.7 *^d,x^*	465 ± 5 *^b,x^*
Syringic acid	24.4 ± 0.2 *^b,x^*	38.9 ± 1.5 *^a,x^*	25.3 ± 1.7 *^b,x^*	8.0 ± 0.7 *^c,x^*	40.5 ± 2.8 *^a,x^*	28.0 ± 0.8 *^b,y^*	28.7 ± 3.8 *^b,y^*	18.8 ± 1.4 *^c,x^*	52.9 ± 2.3 *^a,y^*	47.8 ± 0.5 *^a,x^*
*p*-Coumaric acid	37.0 ± 1.9 *^b,x^*	45.7 ± 1.0 *^a,x^*	24.0 ± 0.6 *^c,x^*	44.7 ± 2.0 *^a,x^*	39.1 ± 0.5 *^b,x^*	38.0 ± 2.0 *^bc,x^*	43.2 ± 0.3 *^a,y^*	21.4 ± 1.1 *^d,x^*	40.3 ± 1.4 *^ab,x^*	34.2 ± 0.3 *^c,y^*
Ferulic acid	18.3 ± 1.6 *^cd,x^*	20.9 ± 0.9 *^c,x^*	11.0 ± 1.0 *^d,x^*	132 ± 4 *^a,x^*	34.0 ± 0. 8 *^b,x^*	16.4 ± 0.1 *^cd,x^*	21.5 ± 1.0 *^bc,x^*	11.1 ± 0.5 *^d,x^*	118 ± 3 *^a,x^*	26.0 ± 0.2 *^b,x^*
Hesperidin	9513 ± 79 *^d,x^*	13450 ± 211 *^b,x^*	19174 ± 32 *^a,x^*	142 ± 3 *^e,x^*	11768 ± 36 *^c,x^*	8741 ± 99 *^d,y^*	13544 ± 67 *^b,x^*	17220 ± 268 *^a,y^*	76.3 ± 4.9 *^c,y^*	17400 ± 279 *^a,y^*
Hyperoside	331 ± 4 *^e,x^*	1630 ± 20 *^a,x^*	1256 ± 12 *^c,x^*	1021 ± 10 *^d,x^*	1375 ± 3 *^b,x^*	188 ± 3 *^e,y^*	1630 ± 10 *^a,x^*	1101 ± 6 *^c,y^*	847 ± 2 *^d,y^*	1542 ± 29 *^b,y^*
Quercetin	25.9 ± 0.1 *^d,x^*	103 ± 1 *^a,x^*	75.2 ± 2.7 *^c,x^*	13.2 ± 0.4 *^e,x^*	85.6 ± 0.2 *^b,x^*	15.1 ± 0.1 *^d,y^*	179 ± 3 *^a,y^*	43.4 ± 0.3 *^c,y^*	4.20 ± 0.02 *^e,y^*	50.8 ± 0.6 *^b,u^*

Within the row, the means with different superscript letters (*a*–*e*) are significantly different using Tukey’s test at *p* < 0.05. Within the same row, the means with different superscript letters (*x*,*y*) for different extracts of each part are significantly different using the student *t* test at *p* < 0.05. nd: not detected, Fm: Flower methanol extract, Lm: Leaf methanol extract, Sm: Stem methanol extract, Rm: Root methanol extract, WPm: Whole Plant methanol extract, Fw: Flower water extract, Lw: Leaf water extract, Sw: Stem water extract, Rw: Root water extract, WPw: Whole Plant water extract.

**Table 3:** IC_50_ values of *A. pisidicus* extracts.

Cell	Extract	Hours	IC_50_ (µg/mL)	Cell	Extract	Hours	IC_50_ (µg/mL)
**A549**	Rm	24 h	307.160	**HT29**	Lm	24 h	162.967
		72 h	379.765		Rm	24 h	14.89
	Lm	24 h	207.257		Fm	24 h	30.323
**MCF7**	Rm	24 h	540.998		WPm	24 h	292.245
	Lm	24 h	32.057		Lw	24 h	258.594
	Fm	24 h	233.635			48 h	28.640
		48 h	440.874	**H1299**	Fm	24 h	9.57
	Lw	24 h	972.290		Lw	24 h	242.609
		48 h	998.651		Fw	24 h	25.836
	Fw	24 h	173.904	**PANC1**	Rm	24 h	308.23
		48 h	174.745		Sm	24 h	9.57
**22RV1**	Lm	24 h	249.059		Sw	24 h	120.622
	Rm	24 h	150.628				
	Fm	24 h	64.25				
		48 h	1048.57				
	Fw	24 h	646.430				

Fm: Flower methanol extract, Lm: Leaf methanol extract, Sm: Stem methanol extract, Rm: Root methanol extract, WPm: Whole Plant methanol extract, Fw: Flower water extract, Lw: Leaf water extract, Sw: Stem water extract, Rw: Root water extract, WPw: Whole plant water extract.

**Table 4:** Antimicrobial activity results of methanol extracts of *A. pisidicus* against Gram (+) bacteria according to the disk diffusion test.

Bacteria	Fm (mm)	Lm (mm)	Sm (mm)	Rm (mm)	WPm (mm)	A (mm)	N (mm)
*S. aureus* ATCC 25923	9	8	9	-	10	31 (P)	-
*S. aureus* ATCC 29213	8	8	8	-	9	19 (P)	-
*S. aureus* ATCC 43300	8	8	8	-	9	16 (FOX)	-
*S. epidermidis* ATCC 12228	-	-	-	-	-	21 (VA)	-
*E. faecalis* ATCC 51299	-	-	-	-	8	14 (VA)	-
*E. faecalis* ATCC 29212	-	-	-	-	8	20 (VA)	-
*S. pyogenes* ATCC 19615	11	9	10	-	12	42 (P)	-

A: Antibiotic, N: Negative control, P: Penicillin, FOX: Cefoxitin, VA: Vancomycin.

**Table 5:** TP and TF content, TOAC, and TEAC of A. *pisidicus* extracts.

Extract	TP (mg GAE/g)	TF (mg QE/g)	TOAC (mg AAE/g)	TEAC (µM TE/g)
Fm	97.95 ± 11.53	14.37 ± 2.18	31 ± 1.37	168 ± 5.37
Fw	120.17 ± 7.96	14.37 ± 3.38	51 ± 5.07	154 ± 3.38
Rm	220.84 ± 9.80	38.78 ± 3.62	94 ± 7.14	248 ± 8.31
Rw	235.06 ± 3.45	34.61 ± 2.49	96 ± 8.13	234 ± 3.79
Sm	167.79 ± 10.07	23.54 ± 4.39	68 ± 3.68	201 ± 3.96
Sw	134.17 ± 6.59	20.73 ± 4.71	47 ± 2.19	197 ± 1.67
Lm	336.37 ± 9.998	70.14 ± 4.18	291 ± 4.49	700 9.31
Lw	364.42 ± 10.37	51.46 ± 6.37	294 ± 10.17	649 ± 7.19
WPm	317.68 ± 4.87	51.43 ± 5.07	276 ± 3.86	537 ± 4.13
WPw	304.94 ± 13.23	41. 62 ± 4.91	274 ± 4.89	499 ± 8.07

GAE: Gallic acid equivalent, QE: Quercetin equivalent, AAE: Ascorbic acid equivalent TE: Trolox equivalent, Rm: Root methanol, Rw: Root water, Sm: Stem methanol, Sw: Stem water, Fm: Flower methanol, Fw: Flower water, Lm: Leaf methanol, Lw: Leaf water, WPm: Whole plant methanol, WPw: Whole plant water.

**Table 6:** DPPH radical scavenging effects, FRAP, CUPRAC, and inhibiting lipid peroxidation effect in the β-carotene/linoleic acid system of *A. pisidicus* extracts.

Extract	DPPH (EC 50 µg/mL)	FRAP (mM FeSO_4_/g)	CUPRAC (mg AAE/g)	β-Caroten (% Activity of 1 mg/mL)
Fm	744 ± 9.32	0.34 ± 0.05	22 ± 0.87	37.91 ± 1.34
Fw	768 ± 4.3	0.67 ± 0.06	24 ± 3.61	37.28 ± 3.64
Rm	384 ± 7.34	1.43 ± 0. 09	49 ± 3.42	76.84 ± 2.16
Rw	367 ± 5.63	1.30 ± 0.04	44 ± 4.27	71.37 ± 2.51
Sm	717 ± 15.57	0.87 ± 0.07	34 ± 3.95	48.88 ± 0.48
Sw	709 ± 16.38	0.93 ± 0.03	33 ± 4.10	43.19 ± 1.89
Lm	119 ± 10.11	4.19 ± 0.08	140 ± 7.36	88.47 ± 2.62
Lw	124 ± 8.39	3.67 ± 0.05	128 ± 4.58	91.37 ±1.73
WPm	187 ± 9.80	3.52 ± 0.1	138 ± 2.08	83.63 ± 3.18
WPw	146 ± 10.37	3.34 ± 0.06	125 ± 6.17	81.19 ± 2.85

AAE: Ascorbic acid equivalent, Rm: Root methanol, Rw: Root water, Sm: Stem methanol, Sw: Stem water, Fm: Flower methanol, Fw: Flower water, Lm: Leaf methanol, Lw: Leaf water, WPm: Whole plant methanol, WPw: Whole plant water.

**Table 7:** The binding energy of the selected compounds (kcal/mol) and their unique identifiers in PubChem.

Compound	Binding Affinity (kcal/mol)		PubChem CID	
	MDM2	XIAP		
Hesperidin	−7.2	−6.6	10621	
Quercetin	−6.9	−6.3	5280343	
Hyperoside	−6.8	−5.6	5281643	
Catechin	−6.7	−6.4	9064	
Chlorogenic acid	−6.6	−6.7	1794427	
Ferulic acid	−5.5	−5.5	445858	
*p*-Coumaric acid	−5.4	−4.9	637542	
Gentisic acid	−5.0	−4.9	3469	
3-Hydroxybenzoic acid	−5.0	−4.5	7420	
Syringic acid	−4.9	−4.8	10742	
Gallic acid	−4.7	−4.6	370	
Protocatechuic acid	−4.7	−4.5	72	
*p*-Hydroxybenzoic acid	−4.6	−4.4	135	
Co-crystallized ligands	−8.6 ^a^	−8.4 ^b^	56591324 ^a^	134611691 ^b^

^a^: 0R2; ^b^: A4E.

**Table 8:** ESI–MS/MS parameters and analytical characteristics for the analysis of target analytes by MRM negative and positive ionization mode.

Target Compounds	*R*_t_ (min)	Precursor Ion	MRM1 (CE, V)	MRM2 (CE, V)
Compounds analyzed by NI mode				
Gallic acid	8.891	168.9 [M − H]−	125.0 (10)	–
Protocatechuic acid	10.818	152.9 [M − H]−	108.9 (12)	–
3,4-Dihydroxyphenylacetic acid	11.224	167.0 [M − H]−	123.0 (2)	–
(+)-Catechin	11.369	289.0 [M − H]−	245.0 (6)	202.9 (12)
Pyrocatechol	11.506	109.0 [M − H]−	90.6 (18)	52.9 (16)
2,5-Dihydroxybenzoic acid	12.412	152.9 [M − H]−	109.0 (10)	–
4-Hydroxybenzoic acid	12.439	136.9 [M − H]−	93.1 (14)	–
Caffeic acid	12.841	179.0 [M − H]−	135.0 (12)	–
Syringic acid	12.963	196.9 [M − H]−	181.9 (8)	152.8 (6)
3-Hydroxybenzoic acid	13.259	137.0 [M − H]−	93.0 (6)	–
Vanillin	13.397	151.0 [M − H]−	136.0 (10)	–
Verbascoside	13.589	623.0 [M − H]−	461.0 (26)	160.8 (36)
Taxifolin	13.909	303.0 [M − H]−	285.1 (2)	125.0 (14)
Sinapic acid	13.992	222.9 [M − H]−	207.9 (6)	163.8 (6)
*p*-Coumaric acid	14.022	162.9 [M − H]−	119.0 (12)	–
Ferulic acid	14.120	193.0 [M − H]−	177.8 (8)	134.0 (12)
Luteolin 7-glucoside	14.266	447.1 [M − H]−	285.0 (24)	–
Rosmarinic acid	14.600	359.0 [M − H]−	196.9 (10)	160.9 (10)
2-Hydroxycinnamic acid	15.031	162.9 [M − H]−	119.1 (10)	–
Pinoresinol	15.118	357.0 [M − H]−	151.0 (12)	135.7 (34)
Eriodictyol	15.247	287.0 [M − H]−	151.0 (4)	134.9 (22)
Quercetin	15.668	301.0 [M − H]−	178.6 (10)	151.0 (16)
Kaempferol	16.236	285.0 [M − H]−	242.8 (16)	229.1 (18)
Compounds analyzed by PI mode				
Chlorogenic acid	11.802	355.0 [M + H]+	163.0 (10)	–
(−)-Epicatechin	12.458	291.0 [M + H]+	139.1 (12)	122.9 (36)
Hesperidin	14.412	611.1 [M + H]+	449.2 (4)	303.0 (20)
Hyperoside	14.506	465.1 [M + H]+	303.1 (8)	–
Apigenin 7-glucoside	14.781	433.1 [M + H]+	271.0 (18)	–
Luteolin	15.923	287.0 [M + H]+	153.1 (34)	135.1 (36)
Apigenin	16.382	271.0 [M + H]+	153.0 (34)	119.1 (36)

*R*_t_, retention time; NI, negative ion; PI, positive ion; MRM, multiple reaction monitoring; CE, collision energy; V, voltage.

**Table 9:** Calibration curves and sensitivity properties of the method.

	Linearity and Sensitivity Characteristics				
Compounds	Range (μg/L)	Linear Equation	R2	LOD (μg/L)	LOQ (μg/L)
Gallic acid	5–500	y = 4.82x − 26.48	0.9988	1.46	4.88
Protocatechuic acid	2.5–500	y = 5.65x − 9.99	0.9990	1.17	3.88
3,4-Dihydroxyphenylacetic acid	5–500	y = 5.13x − 12.39	0.9990	1.35	4.51
(+)-Catechin	10–500	y = 1.45x + 1.95	0.9974	3.96	13.20
Pyrocatechol	25–400	y = 0.11x − 0.52	0.9916	9.62	32.08
Chlorogenic acid	1–500	y = 12.14x + 32.34	0.9995	0.55	1.82
2,5-Dihydroxybenzoic acid	5–500	y = 3.79x − 14.12	0.9980	2.12	7.08
4-Hydroxybenzoic acid	5–500	y = 7.62x + 22.79	0.9996	1.72	5.72
(−)-Epicatechin	5–500	y = 9.11x − 9.99	0.9971	1.85	6.18
Caffeic acid	5–500	y = 11.09x + 16.73	0.9997	3.15	10.50
Syringic acid	10–500	y = 0.74x − 1.54	0.9975	3.75	12.50
3-Hydroxybenzoic acid	5–500	y = 3.69x − 12.29	0.9991	1.86	6.20
Vanillin	50–500	y = 2.02x + 135.49	0.9926	15.23	50.77
Verbascoside	2.5–500	y = 8.59x − 28.05	0.9988	0.82	2.75
Taxifolin	5–500	y = 12.32x + 9.98	0.9993	1.82	6.05
Sinapic acid	5–500	y = 2.09x − 6.79	0.9974	2.64	8.78
*p*-Coumaric acid	5–500	y = 17.51x + 53.73	0.9997	1.93	6.44
Ferulic acid	5–500	y = 3.32x − 4.30	0.9992	1.43	4.76
Luteolin 7-glucoside	1–500	y = 45.25x + 156.48	0.9996	0.45	1.51
Hesperidin	5–500	y = 5.98x + 0.42	0.9993	1.73	5.77
Hyperoside	2.5–500	y = 16.32x − 1.26	0.9998	0.99	3.31
Rosmarinic acid	1–500	y = 9.82x − 17.98	0.9989	0.57	1.89
Apigenin 7-glucoside	1–500	y = 21.33x − 31.69	0.9983	0.41	1.35
2-Hydroxycinnamic acid	1–500	y = 16.72x − 26.94	0.9996	0.61	2.03
Pinoresinol	10–500	y = 0.80x − 2.69	0.9966	3.94	13.12
Eriodictyol	2.5–500	y = 14.24x − 0.50	0.9998	0.80	2.68
Quercetin	5–500	y = 14.68x − 18.25	0.9997	1.23	4.10
Luteolin	5–500	y = 8.96x + 26.80	0.9992	1.34	4.46
Kaempferol	10–500	y = 0.82x − 3.06	0.9959	3.30	10.99
Apigenin	2.5–500	y = 11.29x + 38.05	0.9987	0.96	3.20

LOD and LOQ: limit of detection and limit of quantification, respectively.

## Data Availability

The original contributions presented in this study are included in the article/Supplementary Materials. Further inquiries can be directed to the corresponding author.

## Supplementary Materials

The following supporting information can be downloaded at: https://www.mdpi.com/article/10.3390/ph18010010/s1, Table S1: Pearson correlation analysis results of methanol extract of *A. pisidicus*. Figure S1: Effects of *A. pisidicus* methanol extract on cell viability in 22RV1 lines. Cell viability was assessed by WST-1 assays, and the results are presented as optical density (OD_450_) values at 450 nm for (A) Fm, (B) Lm, (C) Rm, (D) Sm, and (E) WPm for 24, 48, and 72 h (* *p* < 0.05, ** *p* < 0.01, and *** *p* < 0.001); Figure S2: Effects of *A. pisidicus* water extract on cell viability in 22RV1 lines. Cell viability was assessed by WST-1 assays, and the results are presented as optical density (OD_450_) values at 450 nm for (A) Fw, (B) Lw, (C) Rw, (D) Sw, and (E) WPw for 24, 48, and 72 h (* *p* < 0.05, ** *p* < 0.01, and *** *p* < 0.001); Figure S3: Effects of *A. pisidicus* methanol extract on cell viability in A549 lines. Cell viability was assessed by WST-1 assays, and the results are presented as optical density (OD_450_) values at 450 nm for A) Fm, (B) Lm, (C) Rm, (D) Sm, and (E) WPm for 24, 48, and 72 h (* *p* < 0.05, ** *p* < 0.01, and *** *p* < 0.001); Figure S4: Effects of *A. pisidicus* water extract on cell viability in A549 lines. Cell viability was assessed by WST-1 assays, and the results are presented as optical density (OD_450_) values at 450 nm for (A) Fw, (B) Lw, (C) Rw, (D) Sw, and (E) WPw for 24, 48, and 72 h (* *p* < 0.05, ** *p* < 0.01, and *** *p* < 0.001); Figure S5: Effects of *A. pisidicus* methanol extract on cell viability in H1299 lines. Cell viability was assessed by WST-1 assays, and the results are presented as optical density (OD_450_) values at 450 nm for (A) Fm, (B) Lm, (C) Rm, (D) Sm, and (E) WPm for 24, 48, and 72 h (* *p* < 0.05, ** *p* < 0.01, and *** *p* < 0.001); Figure S6: Effects of *A. pisidicus* water extract on cell viability in H1299 lines. Cell viability was assessed by WST-1 assays, and the results are presented as optical density (OD_450_) values at 450 nm for (A) Fw, (B) Lw, (C) Rw, (D) Sw, and (E) WPw for 24, 48, and 72 h (* *p* < 0.05, ** *p* < 0.01, and *** *p* < 0.001); Figure S7: Effects of *A. pisidicus* methanol extract on cell viability in HeLa lines. Cell viability was assessed by WST-1 assays, and the results are presented as optical density (OD_450_) values at 450 nm for (A) Fm, (B) Lm, (C) Rm, (D) Sm, and (E) WPm for 24, 48, and 72 h (* *p* < 0.05, ** *p* < 0.01, and *** *p* < 0.001); Figure S8: Effects of *A. pisidicus* water extract on cell viability in HeLa lines. Cell viability was assessed by WST-1 assays, and the results are presented as optical density (OD450) values at 450 nm for (A) Fw, (B) Lw, (C) Rw, (D) Sw, and (E) WPw for 24, 48, and 72 h (* *p* < 0.05, ** *p* < 0.01, and *** *p* < 0.001); Figure S9: Effects of *A. pisidicus* methanol extract on cell viability in HT29 lines. Cell viability was assessed by WST-1 assays, and the results are presented as optical density (OD_450_) values at 450 nm for (A) Fm, (B) Lm, (C) Rm, (D) Sm, and (E) WPm for 24, 48, and 72 h (* *p* < 0.05, ** *p* < 0.01, and *** *p* < 0.001); Figure S10: Effects of *A. pisidicus* water extract on cell viability in HT29 lines. Cell viability was assessed by WST-1 assays, and the results are presented as optical density (OD_450_) values at 450 nm for (A) Fw, (B) Lw, (C) Rw, (D) Sw, and (E) WPw for 24, 48, and 72 h (* *p* < 0.05, ** *p* < 0.01, and *** *p* < 0.001); Figure S11: Effects of *A. pisidicus* methanol extract on cell viability in MCF7 lines. Cell viability was assessed by WST-1 assays, and the results are presented as optical density (OD_450_) values at 450 nm for (A) Fm, (B) Lm, (C) Rm, (D) Sm, and (E) WPm for 24, 48, and 72 h (* *p* < 0.05, ** *p* < 0.01, and *** *p* < 0.001); Figure S12: Effects of *A. pisidicus* water extract on cell viability in MCF7 lines. Cell viability was assessed by WST-1 assays, and the results are presented as optical density (OD_450_) values at 450 nm for (A) Fw, (B) Lw, (C) Rw, (D) Sw, and (E) WPw for 24, 48, and 72 h (* *p* < 0.05, ** *p* < 0.01, and *** *p* < 0.001); Figure S13: Effects of *A. pisidicus* methanol extract on cell viability in MDA lines. Cell viability was assessed by WST-1 assays, and the results are presented as optical density (OD_450_) values at 450 nm for (A) Fm, (B) Lm, (C) Rm, (D) Sm, and (E) WPm for 24, 48, and 72 h (* *p* < 0.05, ** *p* < 0.01, and *** *p* < 0.001); Figure S14: Effects of *A. pisidicus* water extract on cell viability in MDA lines. Cell viability was assessed by WST-1 assays, and the results are presented as optical density (OD_450_) values at 450 nm for (A) Fw, (B) Lw, (C) Rw, (D) Sw, and (E) WPw for 24, 48, and 72 h (* *p* < 0.05, ** *p* < 0.01, and *** *p* < 0.001); Figure S15: Effects of *A. pisidicus* methanol extract on cell viability in PANC1 lines. Cell viability was assessed by WST-1 assays, and the results are presented as optical density (OD_450_) values at 450 nm for (A) Fm, (B) Lm, (C) Rm, (D) Sm, and (E) WPm for 24, 48, and 72 h (* *p* < 0.05, ** *p* < 0.01, and *** *p* < 0.001); Figure S16: Effects of *A. pisidicus* water extract on cell viability in PANC1 lines. Cell viability was assessed by WST-1 assays, and the results are presented as optical density (OD_450_) values at 450 nm for (A) Fw, (B) Lw, (C) Rw, (D) Sw, and (E) WPw for 24, 48, and 72 h (* *p* < 0.05, ** *p* < 0.01, and *** *p* < 0.001); Figure S17: Effects of *A. pisidicus* methanol extract on cell viability in 293T lines. Cell viability was assessed by WST-1 assays, and the results are presented as optical density (OD_450_) values at 450 nm for (A) Fm, (B) Lm, (C) Rm, (D) Sm, and (E) WPm for 24, 48, and 72 h (* *p* < 0.05, ** *p* < 0.01, and *** *p* < 0.001); Figure S18: Effects of *A. pisidicus* water extract on cell viability in 293T lines. Cell viability was assessed by WST-1 assays, and the results are presented as optical density (OD_450_) values at 450 nm for (A) Fw, (B) Lw, (C) Rw, (D) Sw, and (E) WPw for 24, 48, and 72 h (* *p* < 0.05, ** *p* < 0.01, and *** *p* < 0.001).
