# Supplementary material for: Phytochemical and Biological Investigations of Crude Extracts of Astragalus pisidicus
Source: Pharmaceuticals (Basel). 2024 Dec 25;18(1):10. doi: 10.3390/ph18010010 (PMC11768461; doi:10.3390/ph18010010)
Supplement: Supplementary file 1 [file pharmaceuticals-18-00010-s001.zip › pharmaceuticals-3314560-supplementary.pdf]

Supplementary File

Phytochemical and biological investigations of crude extracts of *Astragalus pisidicus*

Esra Aydemir[1*], Elif Odabaş Köse[2], Serap Özkaya Gül[1], Alaaddin Korkut[1], A. Cansu Kilit[3], Mehmet Engin Celep[4], Mustafa Yavuz[1], R. Süleyman Göktürk[1] and Cengiz Sarikurkcu[5]

[1] Department of Biology, Faculty of Science, Akdeniz University, Antalya TR-07058, Turkey
[2] Medical Laboratory Program, Vocational School of Health Services, Akdeniz University, Antalya TR-07058, Turkey
[3] Department of Electronics and Automation, Biomedical Device Technology Program, Technical Sciences Vocational School, Akdeniz University, Antalya TR-07058, Turkey
[4] Department of Pharmacognosy, Faculty of Pharmacy, Yeditepe University, Atasehir, Istanbul TR-34755, Turkey
[5] Department of Analytical Chemistry, Faculty of Pharmacy, Afyonkarahisar Health Sciences University, Afyonkarahisar TR-03100, Turkey
* Correspondence: esra@akdeniz.edu.tr

Table S1. Pearson correlation analysis results of extract of *A. pisidicus*

Compounds	A549-24h	A549 - 72h	H1299-24h	HT29-24h	HT29-48h	MCF7-48h	22RV1-48h
Gallic acid							
Chlorogenic acid		*p=0.05; r= 0.9320					*p=0.05; r= 0.9124
p-Hydroxybenzoic acid			*p=0.05; r= -0.8401			*p=0.05; r= -0.8801	
3-Hydroxybenzoic acid			*p=0.05; r= -0.8341	*p=0.05; r=-0.7098			
p- coumaric acid							
Ferulic acid			*** p=0.001; r= 0.9840		*** p=0.001; r= 0.9922	* p=0.05; r= 0.8564	
Hesperidin			* p=0.05; r= -0.99			*p=0.05; r= -0.8708	

1. **Cell proliferation (WST-1) assay**

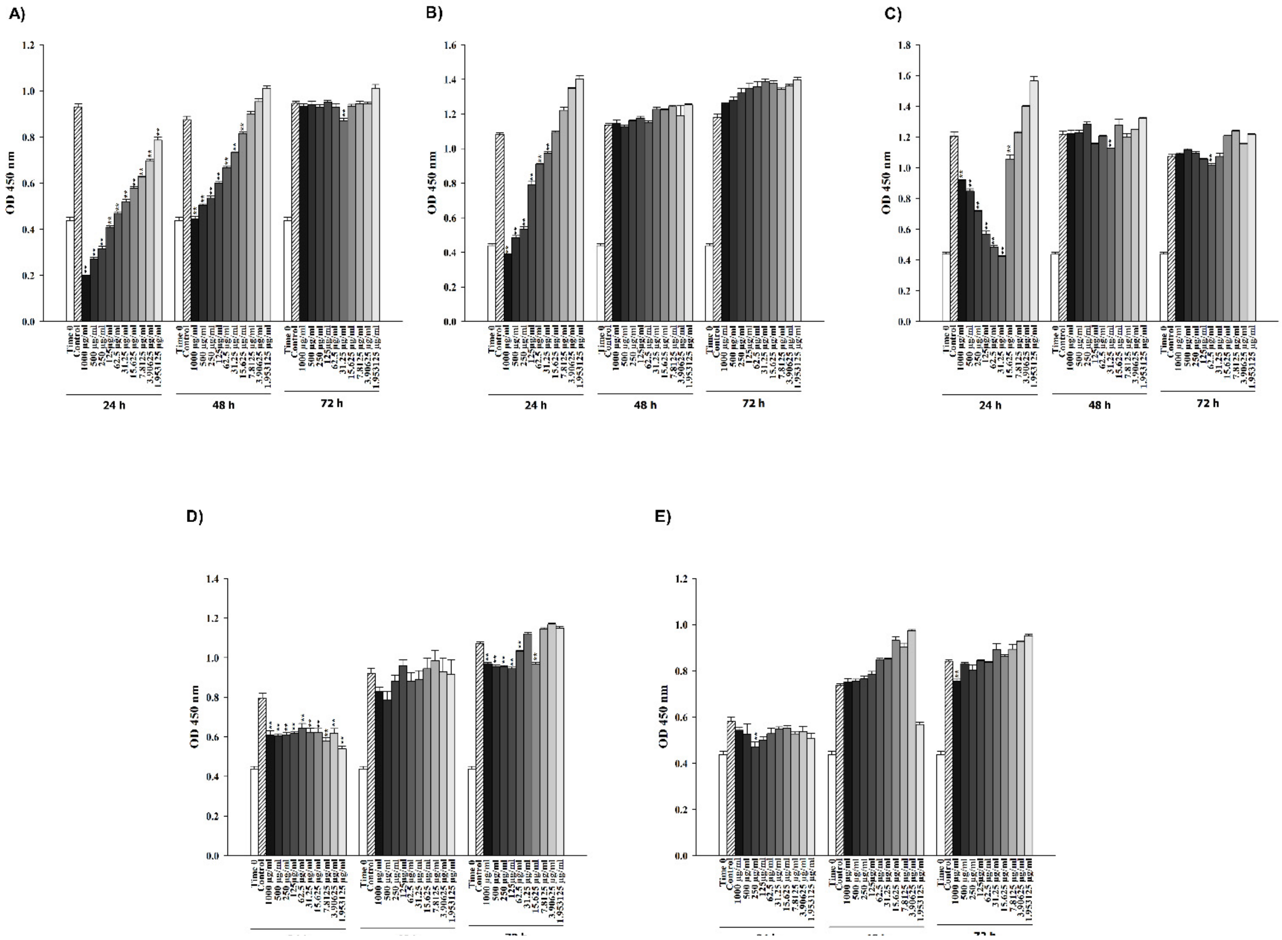

Figure S1: Effects of *A. pisidicus* methanol extract on cell viability in 22RV1 lines. Cell viability was assessed by WST-1 assays and the results are presented as optical density (OD_{450}) values at 450 nm for A) Fm, (B) Lm, (C) Rm, (D) Sm and (E) WPm for 24, 48 and 72 h (* $p < 0.05$, ** $p < 0.01$ and *** $p < 0.001$).

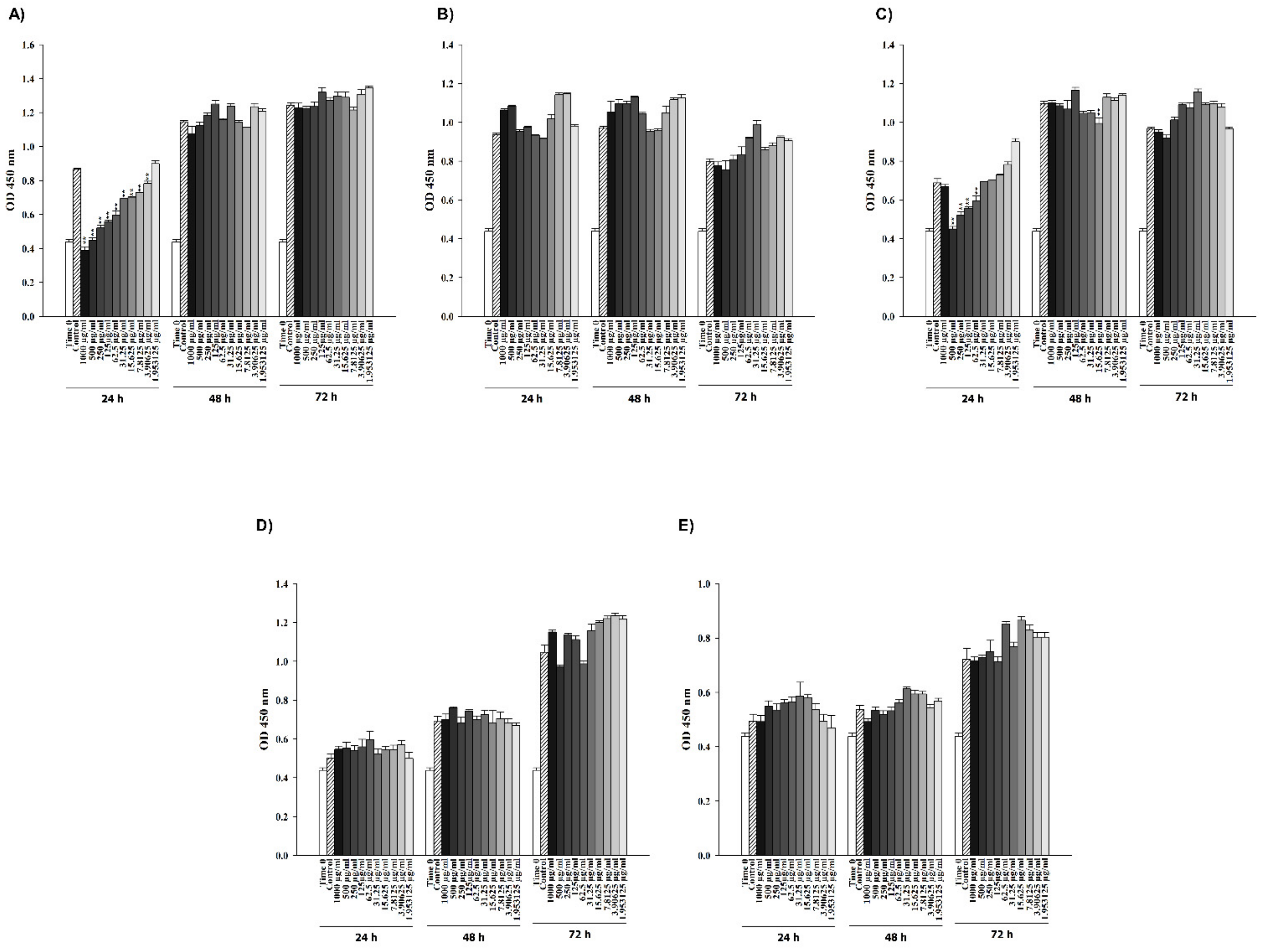

Figure S2: Effects of *A. pisidicus* water extract on cell viability in 22RV1 lines. Cell viability was assessed by WST-1 assays and the results are presented as optical density (OD_{450}) values at 450 nm for A) Fw, (B) Lw, (C) Rw, (D) Sw and (E) WPw for 24, 48 and 72 h (* $p < 0.05$, ** $p < 0.01$ and *** $p < 0.001$).

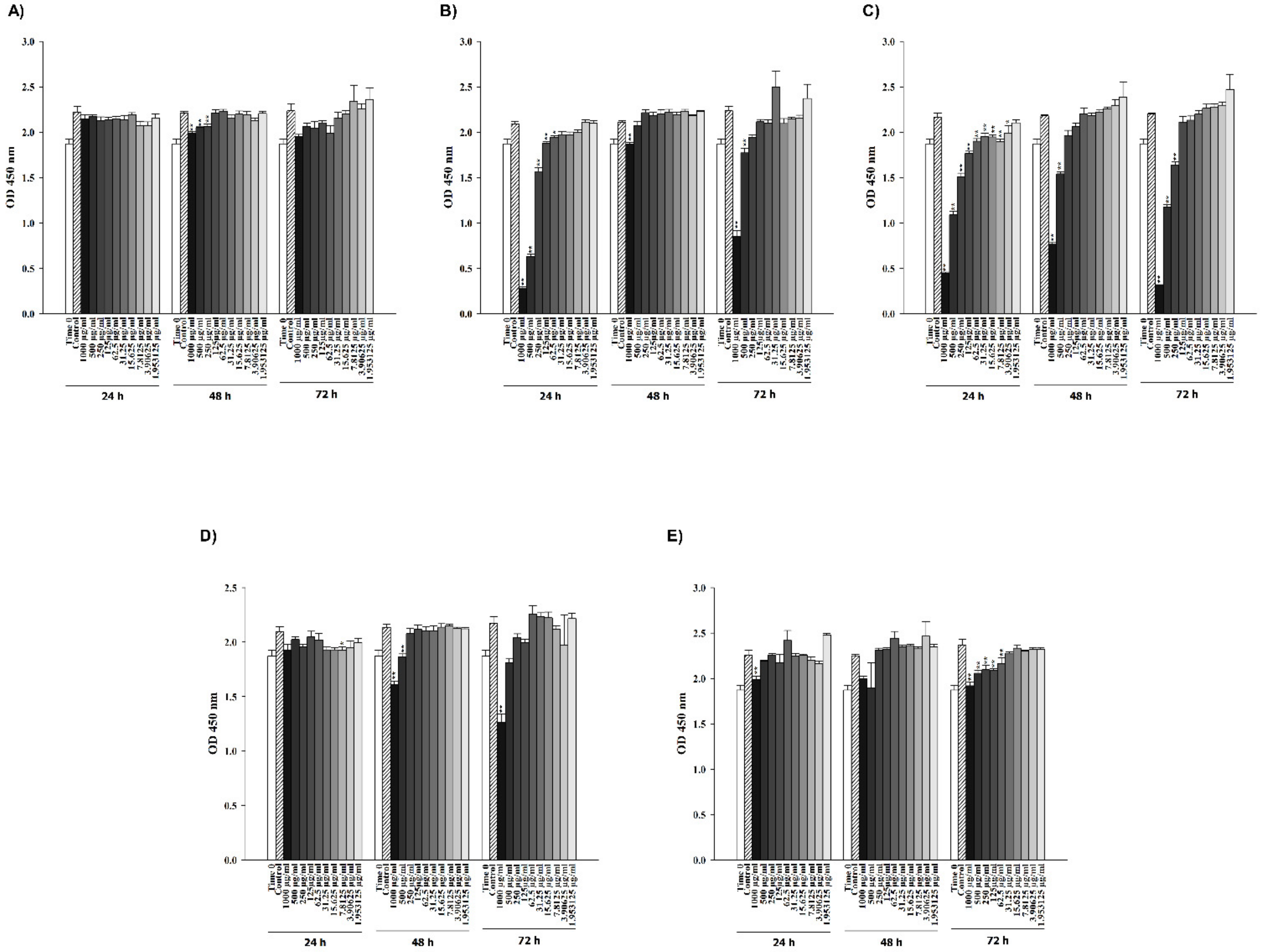

Figure S3: Effects of *A. pisidicus* methanol extract on cell viability in A549 lines. Cell viability was assessed by WST-1 assays and the results are presented as optical density (OD_{450}) values at 450 nm for A) Fm, (B) Lm, (C) Rm, (D) Sm and (E) WPm for 24, 48 and 72 h (* $p < 0.05$, ** $p < 0.01$ and *** $p < 0.001$).

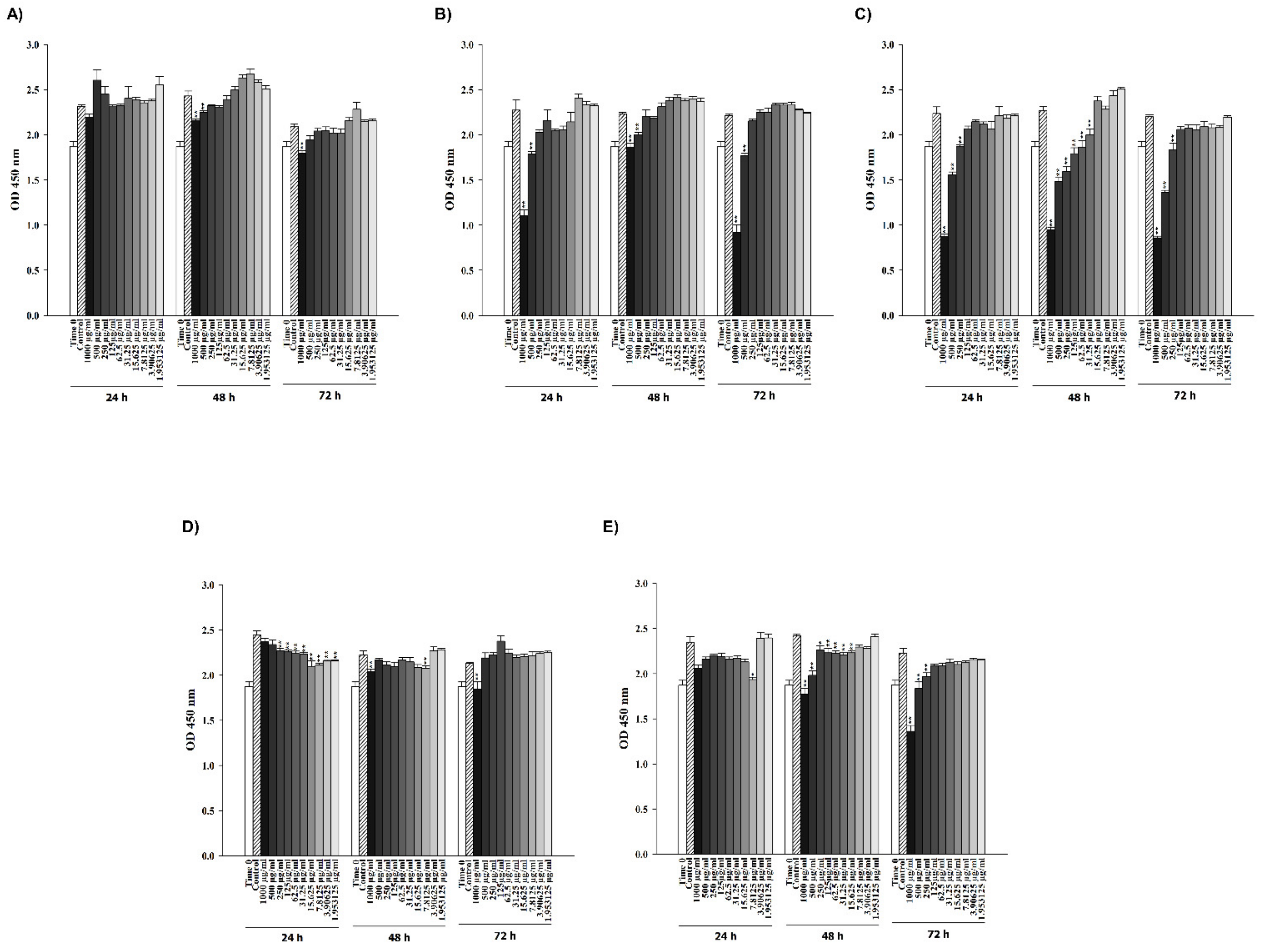

Figure S4: Effects of *A. pisidicus* water extract on cell viability in A549 lines. Cell viability was assessed by WST-1 assays and the results are presented as optical density (OD_{450}) values at 450 nm for A) Fw, (B) Lw, (C) Rw, (D) Sw and (E) WPw for 24, 48 and 72 h (* $p < 0.05$, ** $p < 0.01$ and *** $p < 0.001$).

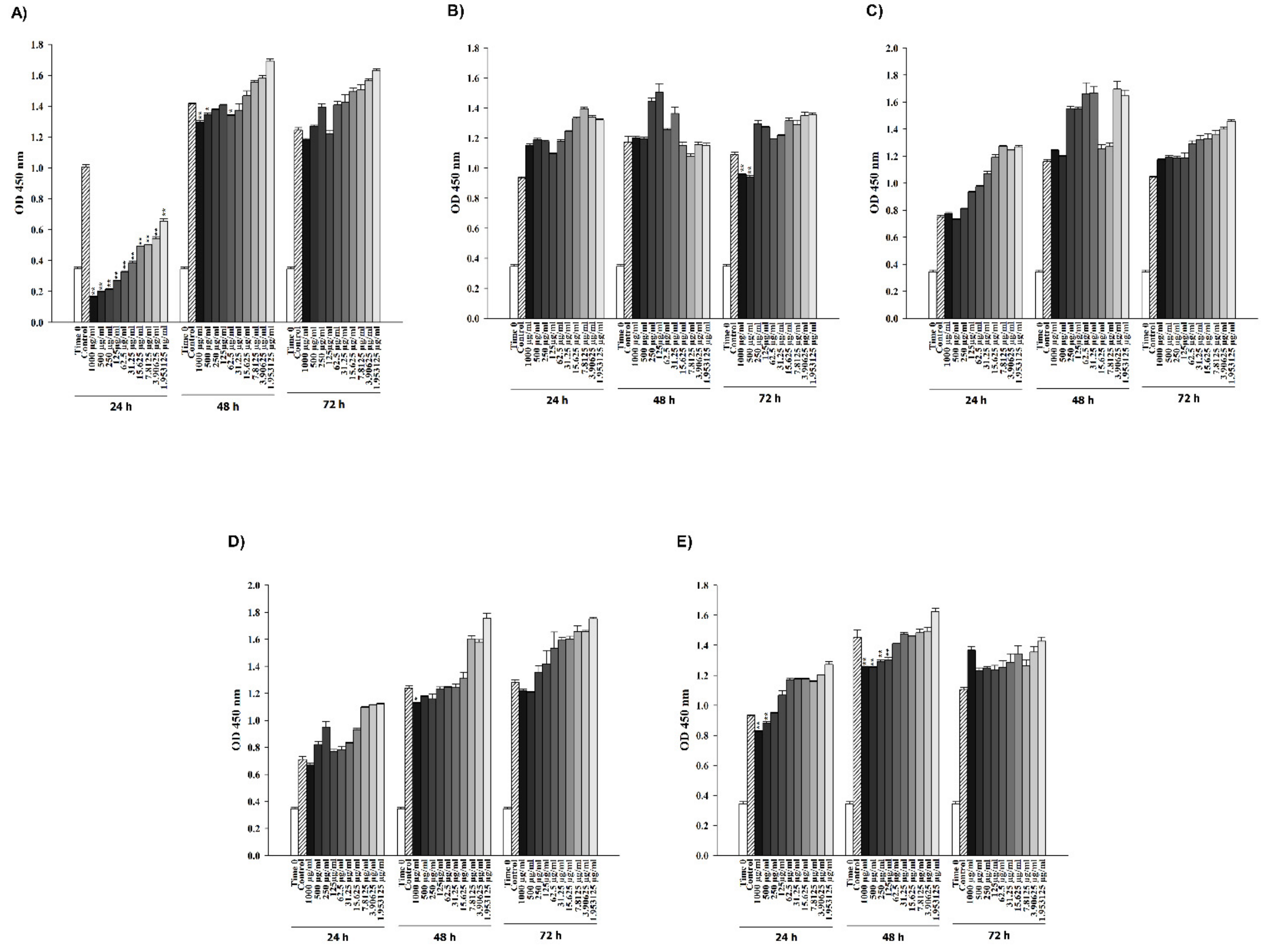

Figure S5: Effects of *A. pisidicus* methanol extract on cell viability in H1299 lines. Cell viability was assessed by WST-1 assays and the results are presented as optical density (OD_{450}) values at 450 nm for A) Fm, (B) Lm, (C) Rm, (D) Sm and (E) WPm for 24, 48 and 72 h (* $p < 0.05$, ** $p < 0.01$ and *** $p < 0.001$).

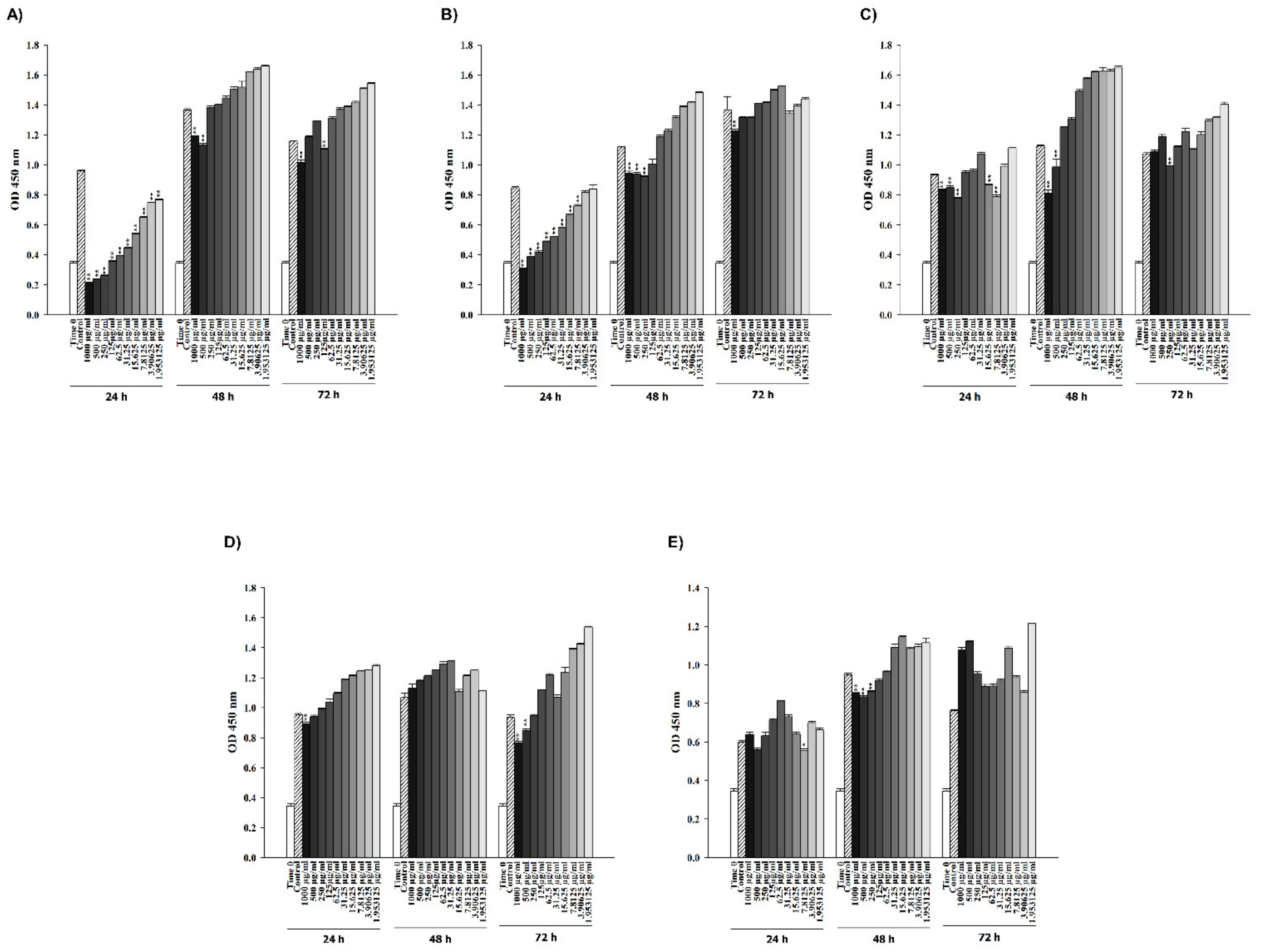

Figure S6: Effects of *A. pisidicus* water extract on cell viability in H1299 lines. Cell viability was assessed by WST-1 assays and the results are presented as optical density (OD_{450}) values at 450 nm for A) Fw, (B) Lw, (C) Rw, (D) Sw and (E) WPw for 24, 48 and 72 h (* $p < 0.05$, ** $p < 0.01$ and *** $p < 0.001$).

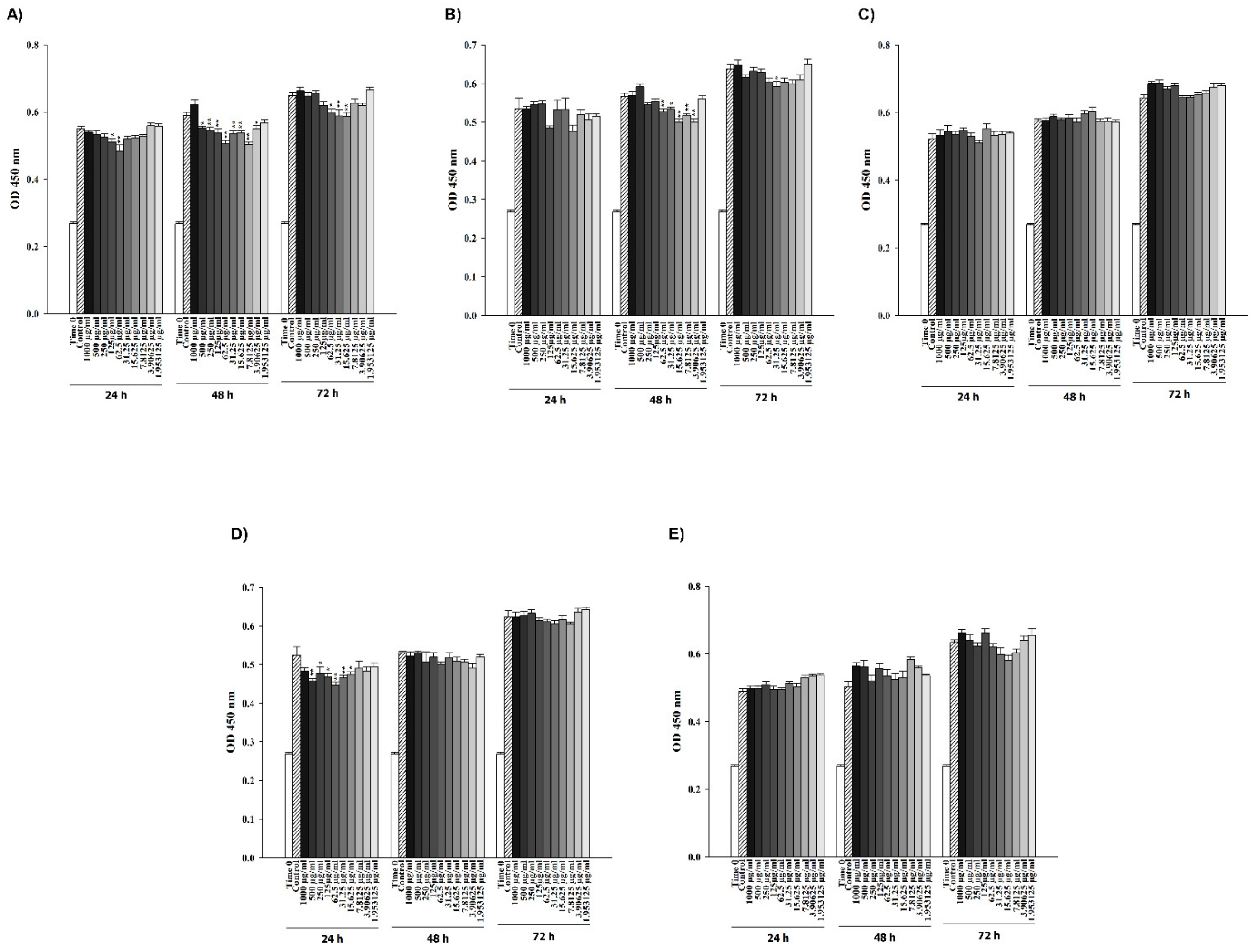

Figure S7: Effects of *A. pisidicus* methanol extract on cell viability in HeLa lines. Cell viability was assessed by WST-1 assays and the results are presented as optical density (OD_{450}) values at 450 nm for A) Fm, (B) Lm, (C) Rm, (D) Sm and (E) WPm for 24, 48 and 72 h (* $p < 0.05$, ** $p < 0.01$ and *** $p < 0.001$).

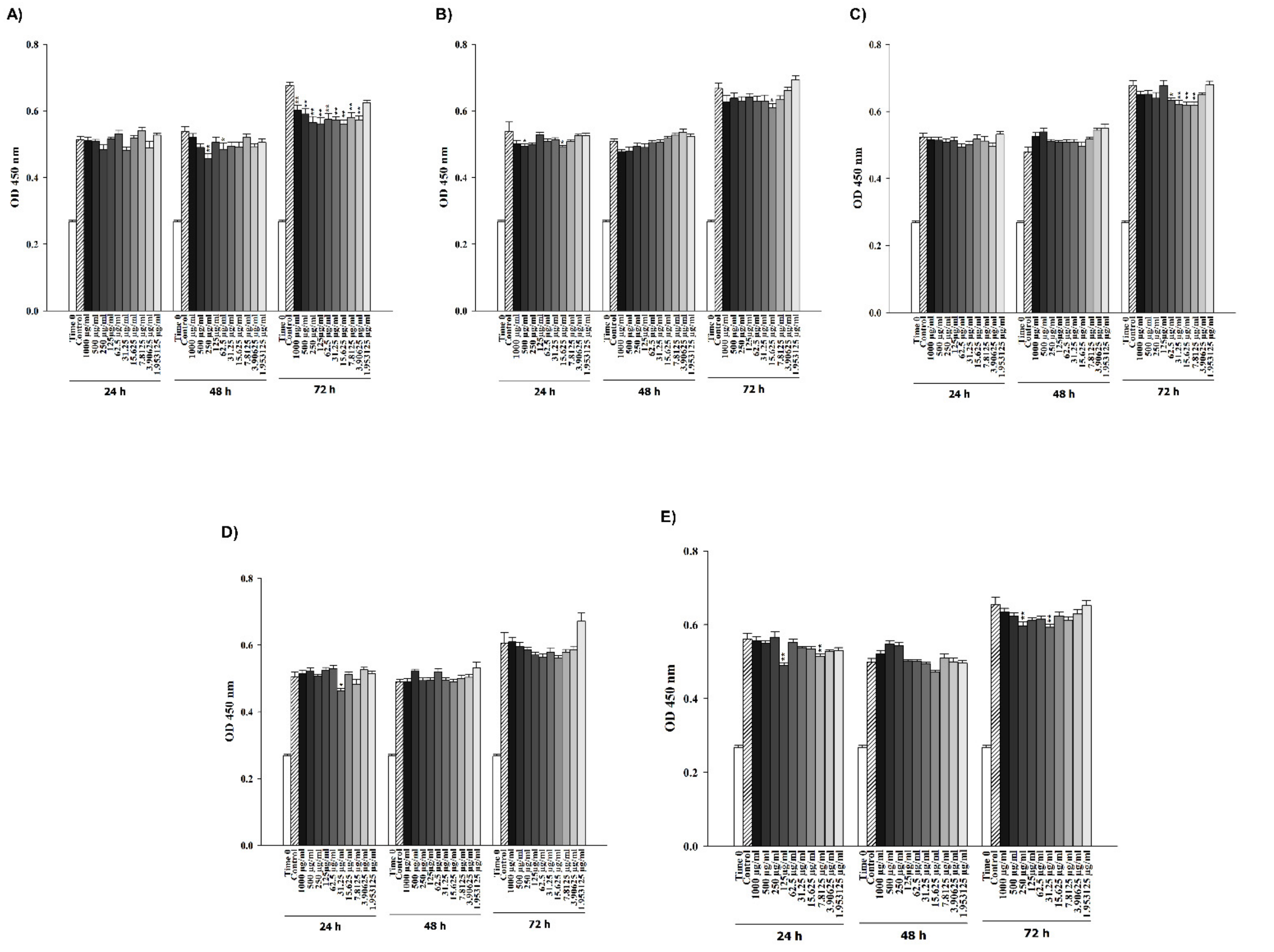

Figure S8: Effects of *A. pisidicus* water extract on cell viability in HeLa lines. Cell viability was assessed by WST-1 assays and the results are presented as optical density (OD_{450}) values at 450 nm for A) Fw, (B) Lw, (C) Rw, (D) Sw and (E) WPw for 24, 48 and 72 h (* $p < 0.05$, ** $p < 0.01$ and *** $p < 0.001$).

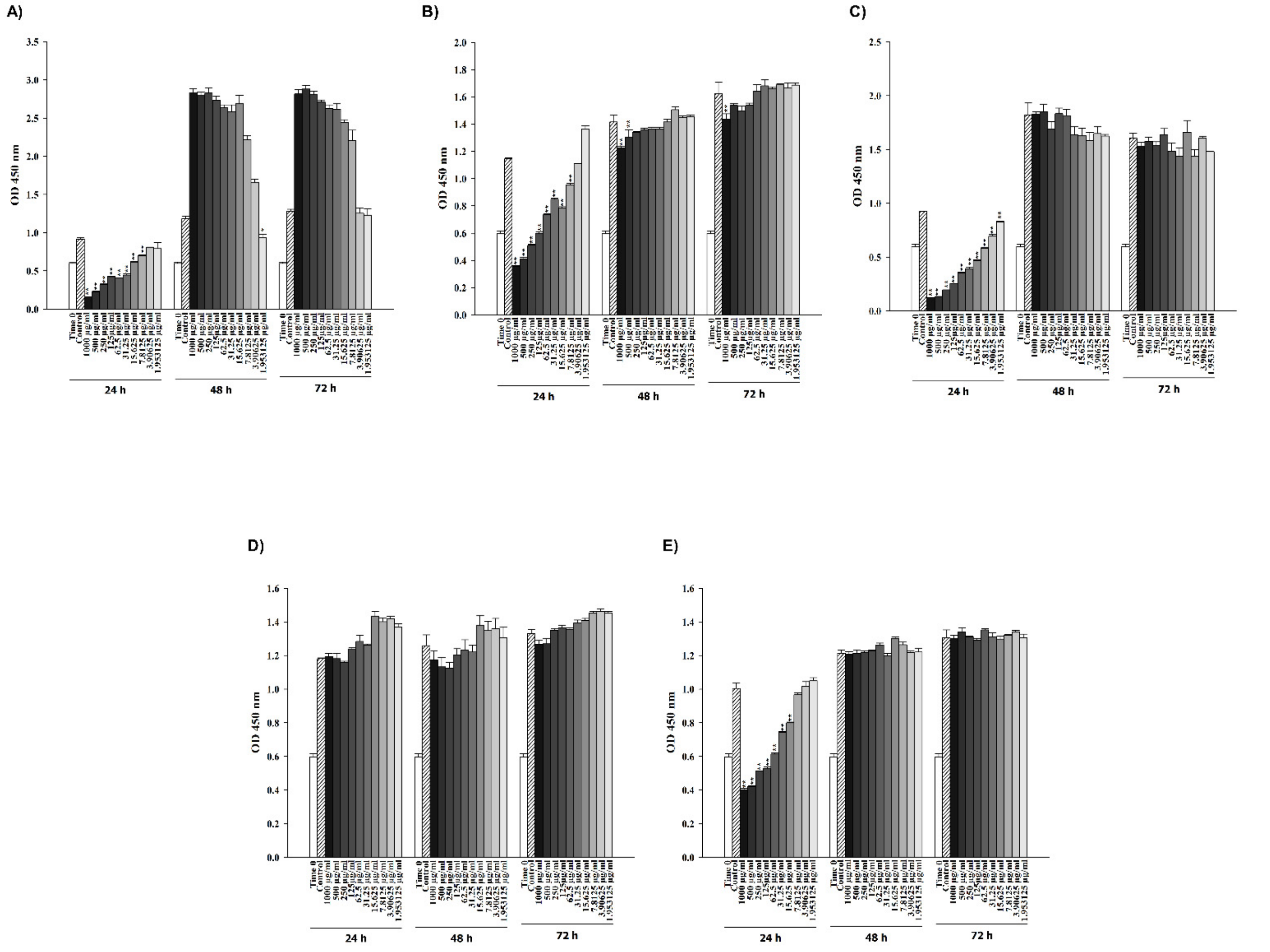

Figure S9: Effects of *A. pisidicus* methanol extract on cell viability in HT29 lines. Cell viability was assessed by WST-1 assays and the results are presented as optical density (OD_{450}) values at 450 nm for A) Fm, (B) Lm, (C) Rm, (D) Sm and (E) WPm for 24, 48 and 72 h (* $p < 0.05$, ** $p < 0.01$ and *** $p < 0.001$).

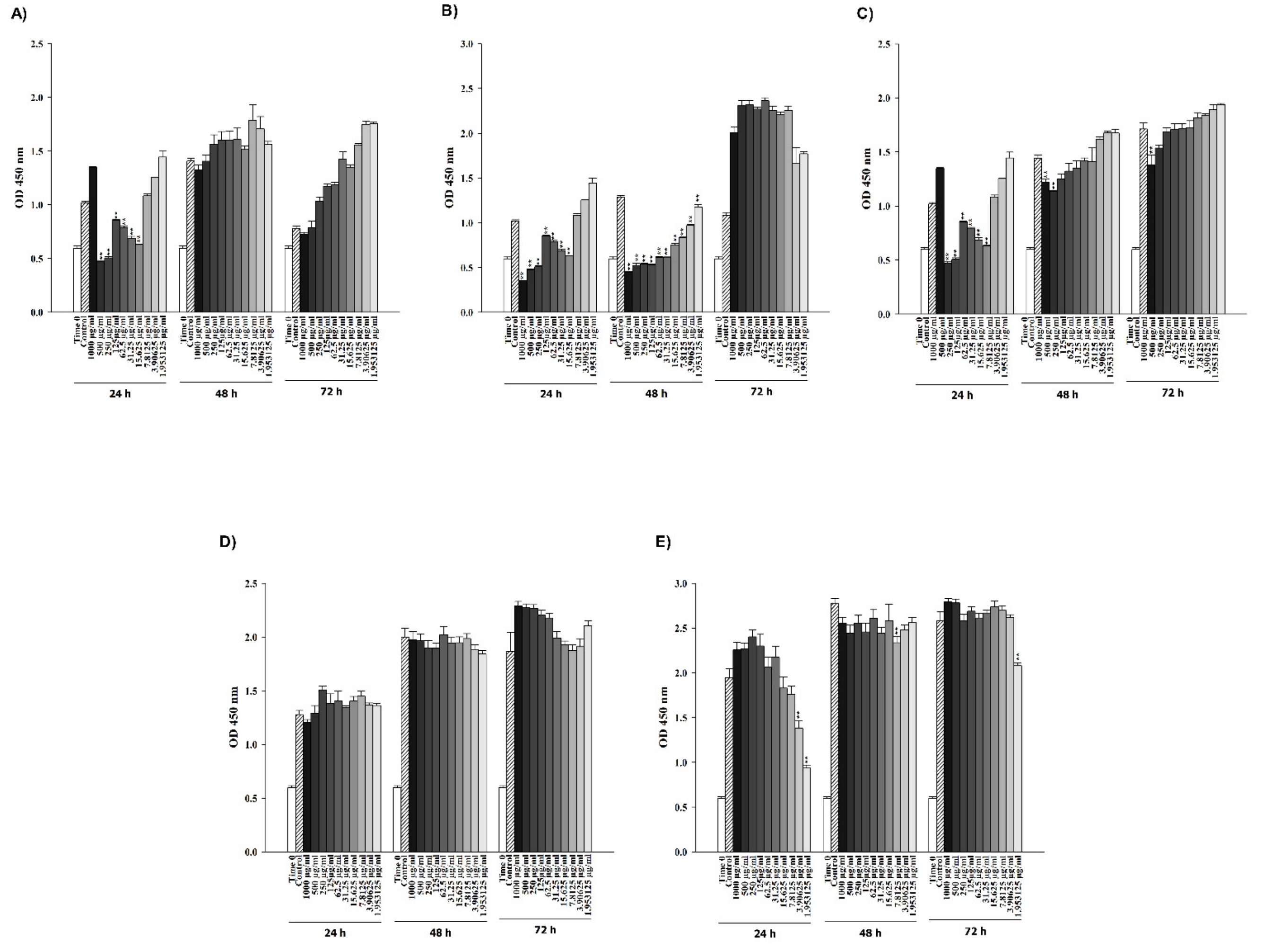

Figure S10: Effects of *A. pisidicus* water extract on cell viability in HT29 lines. Cell viability was assessed by WST-1 assays and the results are presented as optical density (OD_{450}) values at 450 nm for A) Fw, (B) Lw, (C) Rw, (D) Sw and (E) WPw for 24, 48 and 72 h (* $p < 0.05$, ** $p < 0.01$ and *** $p < 0.001$).

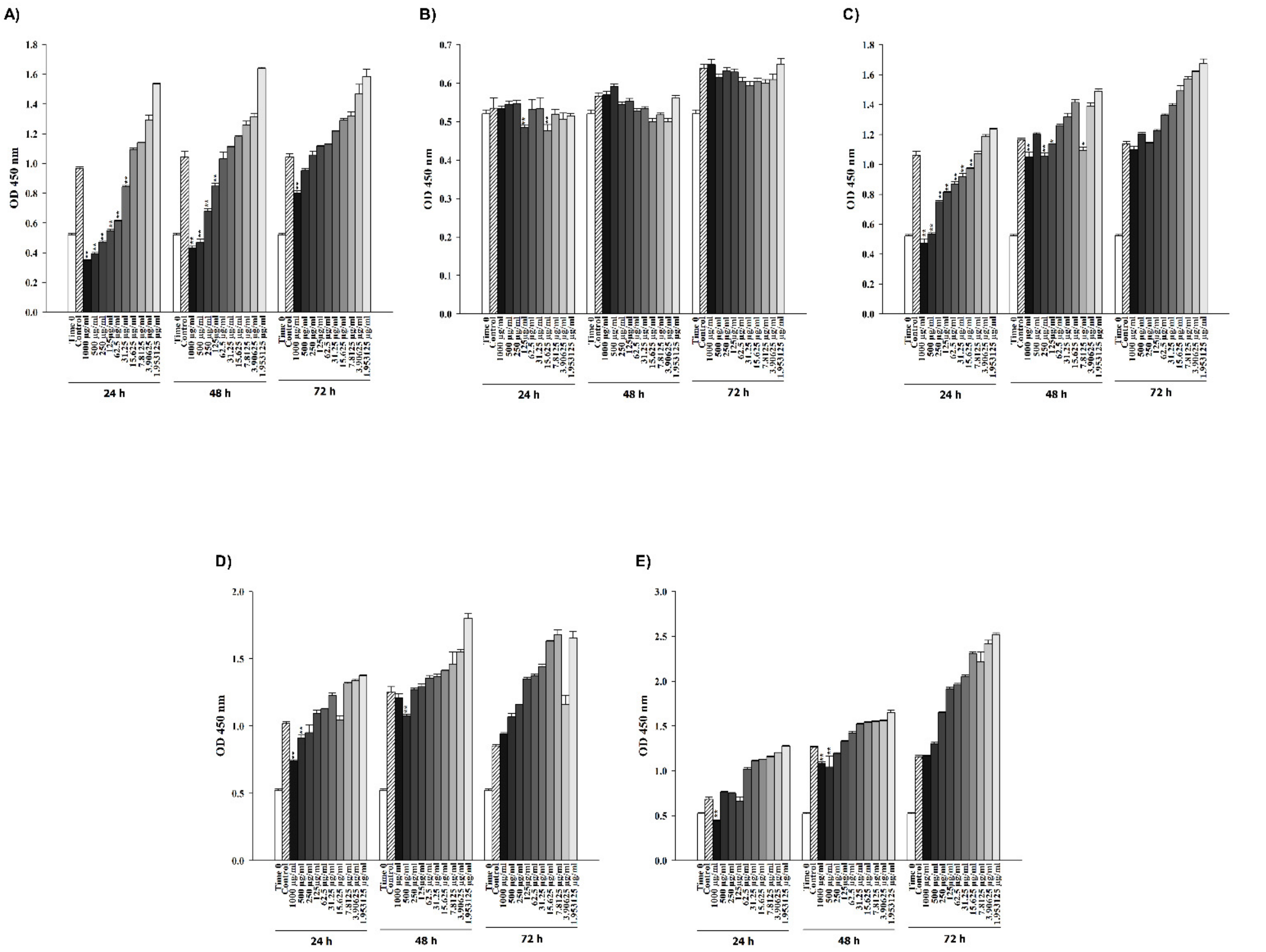

Figure S11: Effects of *A. pisidicus* methanol extract on cell viability in MCF7 lines. Cell viability was assessed by WST-1 assays and the results are presented as optical density (OD_{450}) values at 450 nm for A) Fm, (B) Lm, (C) Rm, (D) Sm and (E) WPm for 24, 48 and 72 h (* $p < 0.05$, ** $p < 0.01$ and *** $p < 0.001$).

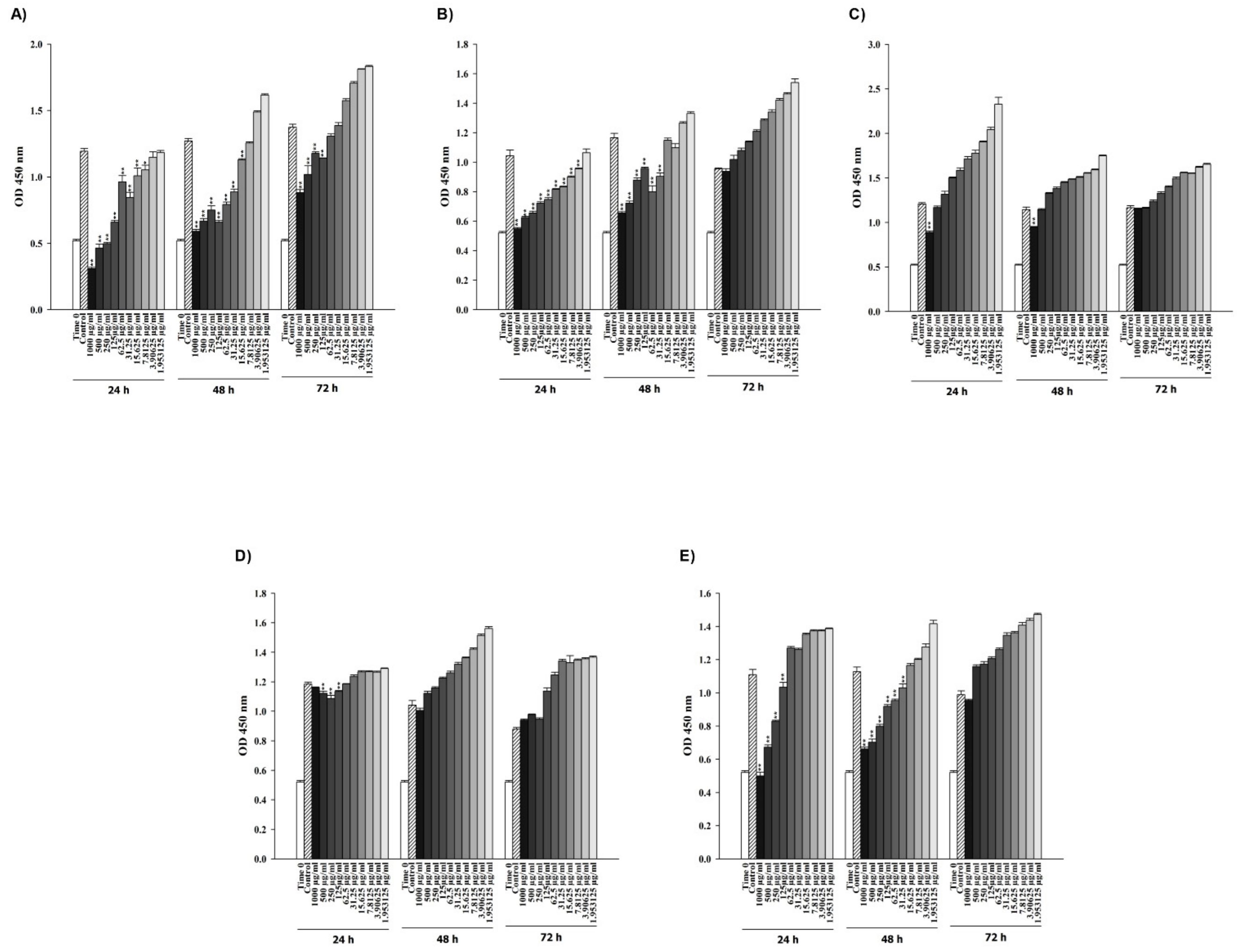

Figure S12: Effects of *A. pisidicus* water extract on cell viability in MCF7 lines. Cell viability was assessed by WST-1 assays and the results are presented as optical density (OD_{450}) values at 450 nm for A) Fw, (B) Lw, (C) Rw, (D) Sw and (E) WPw for 24, 48 and 72 h (* $p < 0.05$, ** $p < 0.01$ and *** $p < 0.001$).

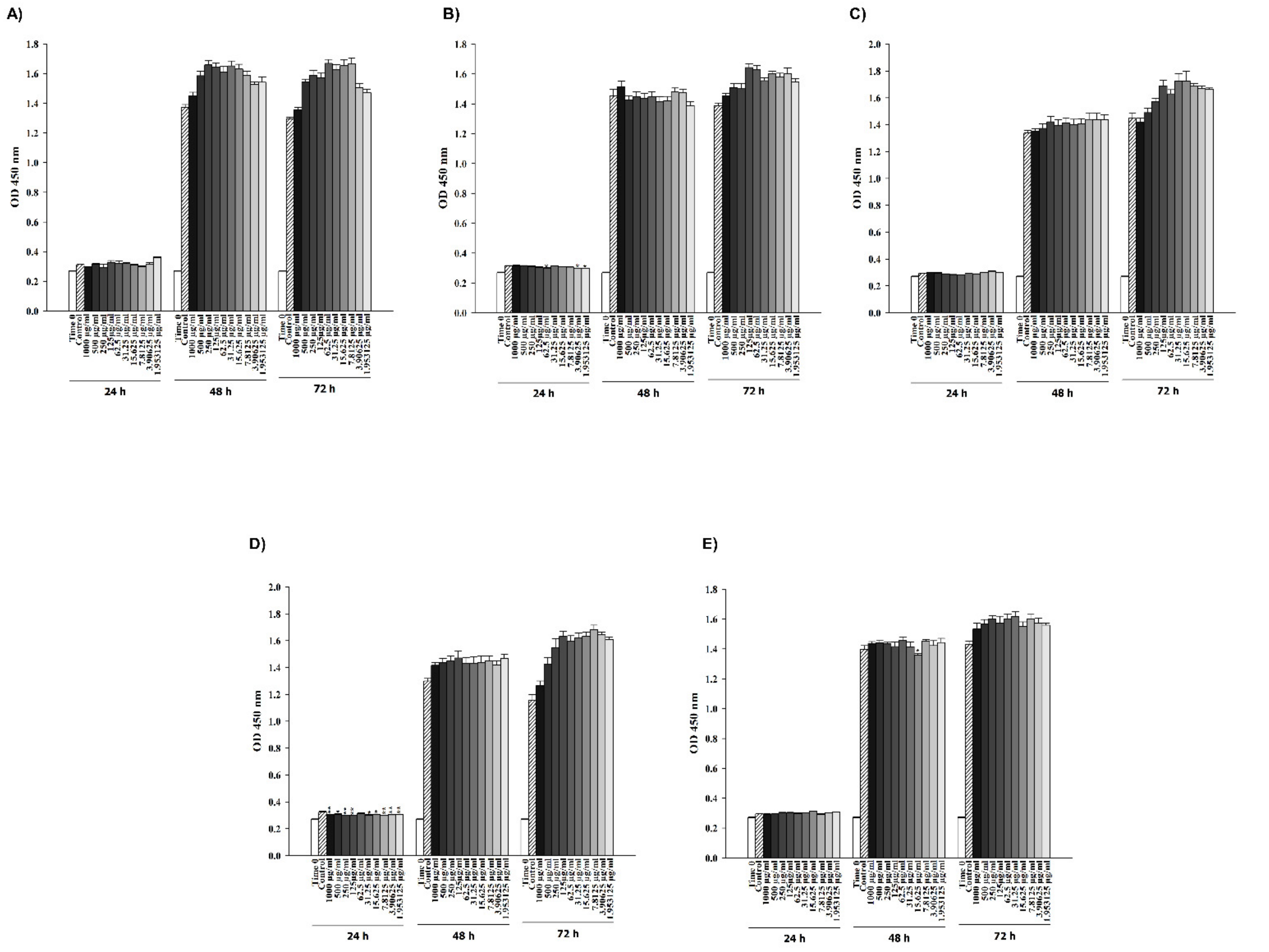

Figure S13: Effects of *A. pisidicus* methanol extract on cell viability in MDA lines. Cell viability was assessed by WST-1 assays and the results are presented as optical density (OD_{450}) values at 450 nm for A) Fm, (B) Lm, (C) Rm, (D) Sm and (E) WPm for 24, 48 and 72 h (* $p < 0.05$, ** $p < 0.01$ and *** $p < 0.001$).

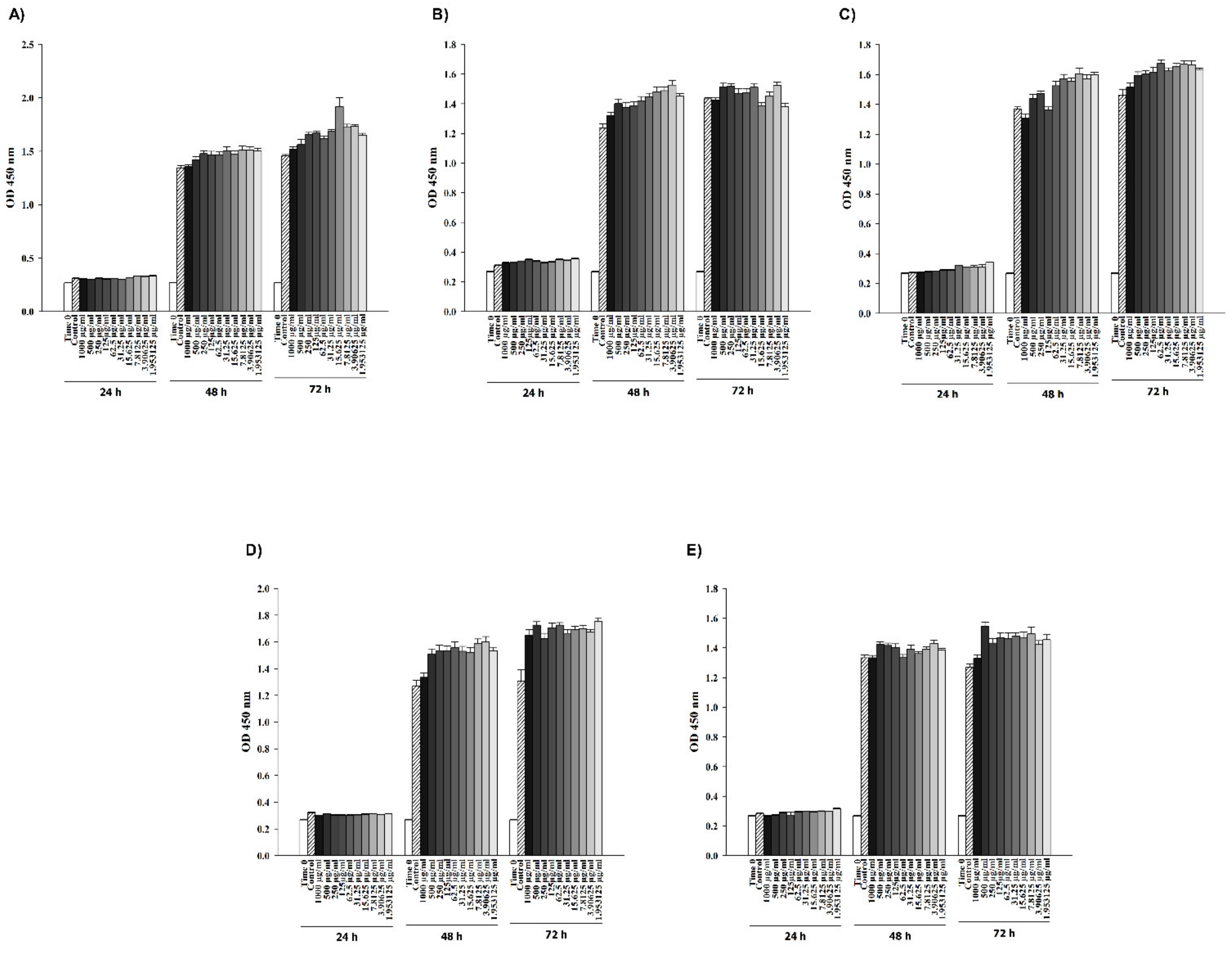

Figure S14: Effects of *A. pisidicus* water extract on cell viability in MDA lines. Cell viability was assessed by WST-1 assays and the results are presented as optical density (OD_{450}) values at 450 nm for A) Fw, (B) Lw, (C) Rw, (D) Sw and (E) WPw for 24, 48 and 72 h (* $p < 0.05$, ** $p < 0.01$ and *** $p < 0.001$).

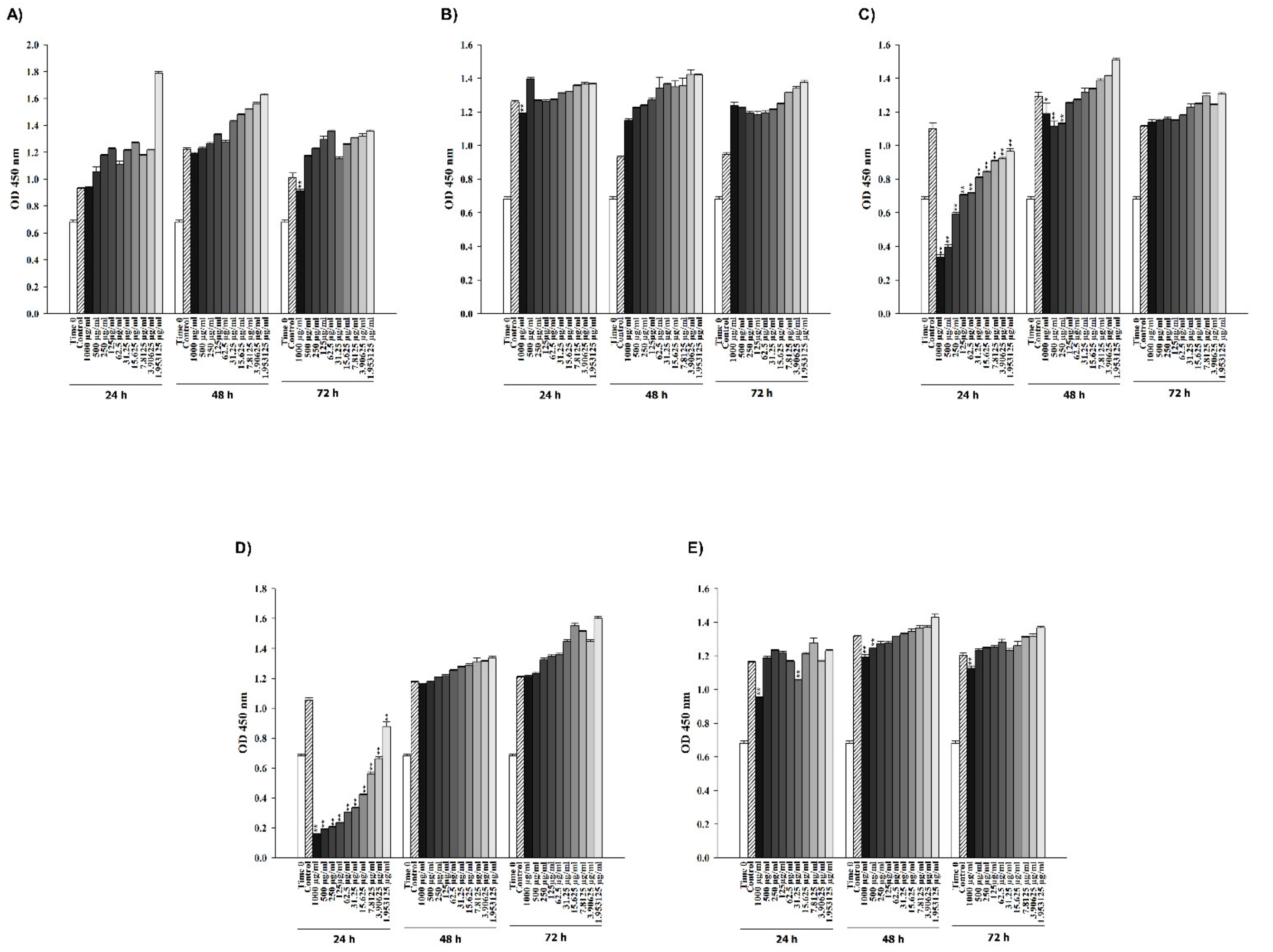

Figure S15: Effects of *A. pisidicus* methanol extract on cell viability in PANC1 lines. Cell viability was assessed by WST-1 assays and the results are presented as optical density (OD_{450}) values at 450 nm for A) Fm, (B) Lm, (C) Rm, (D) Sm and (E) WPm for 24, 48 and 72 h (* $p < 0.05$, ** $p < 0.01$ and *** $p < 0.001$).

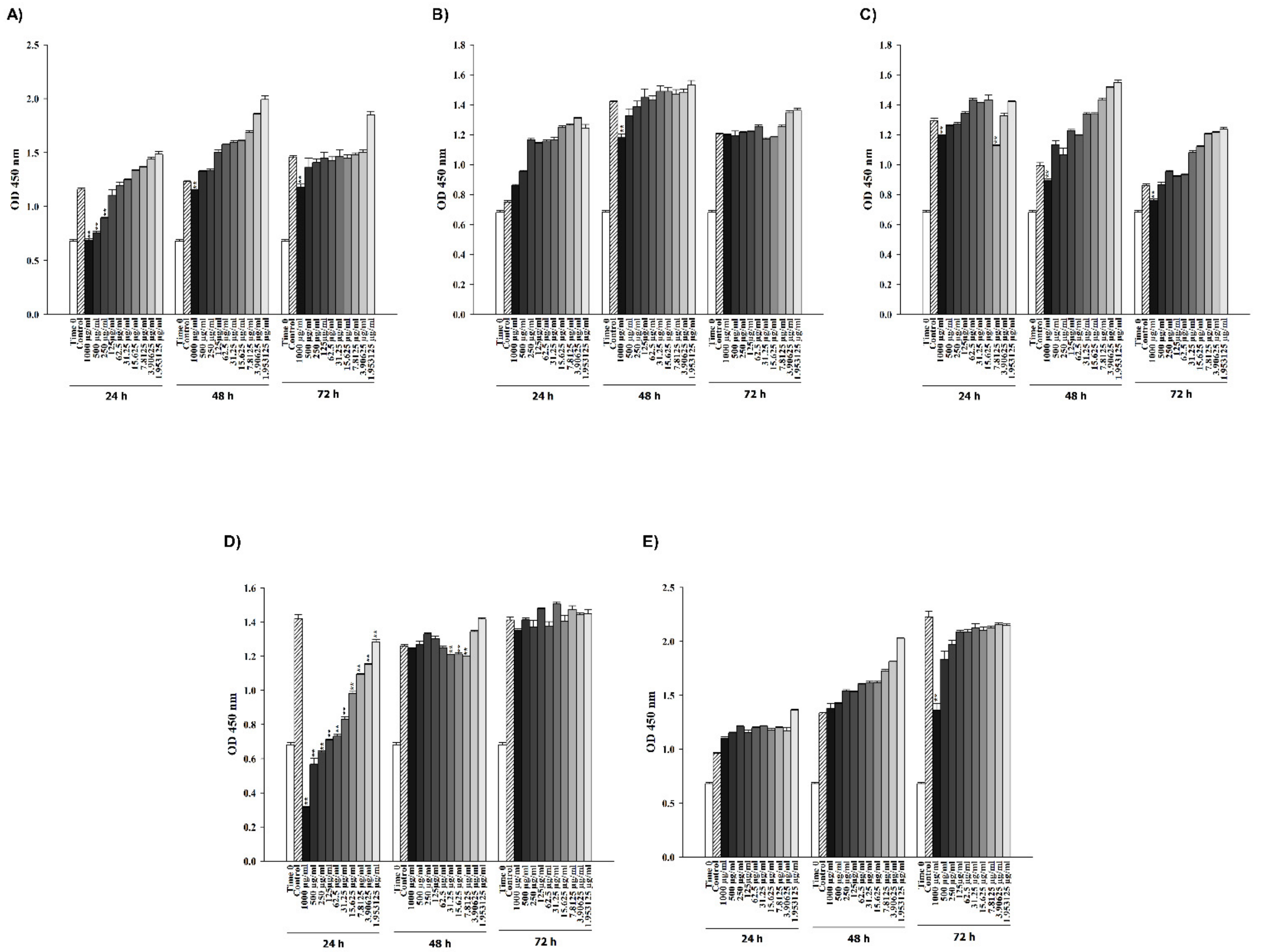

Figure S16: Effects of *A. pisidicus* water extract on cell viability in PANC1 lines. Cell viability was assessed by WST-1 assays and the results are presented as optical density (OD_{450}) values at 450 nm for A) Fw, (B) Lw, (C) Rw, (D) Sw and (E) WPw for 24, 48 and 72 h (* $p < 0.05$, ** $p < 0.01$ and *** $p < 0.001$).

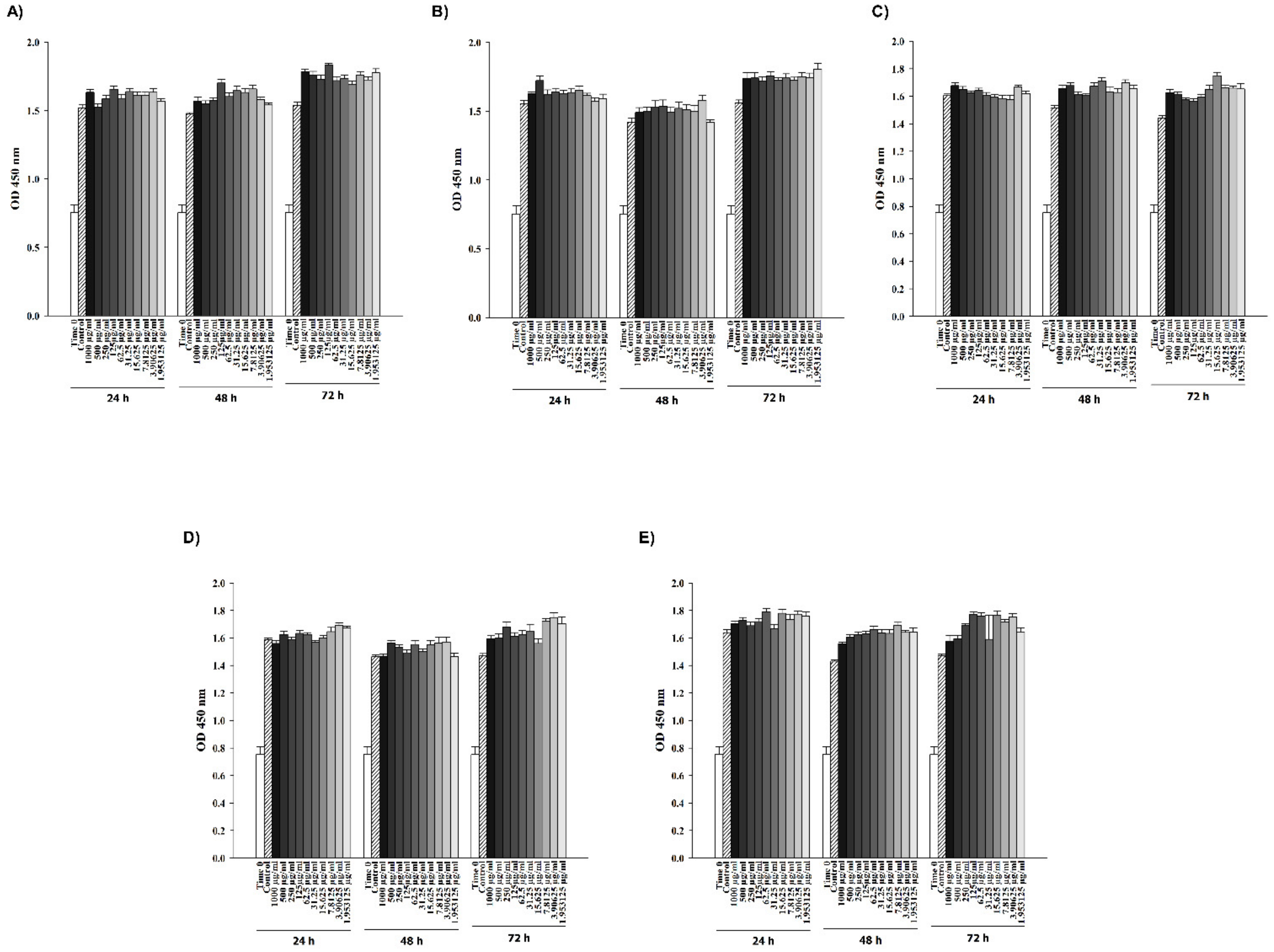

Figure S17: Effects of *A. pisidicus* methanol extract on cell viability in 293T lines. Cell viability was assessed by WST-1 assays and the results are presented as optical density (OD_{450}) values at 450 nm for A) Fm, (B) Lm, (C) Rm, (D) Sm and (E) WPm for 24, 48 and 72 h (* $p < 0.05$, ** $p < 0.01$ and *** $p < 0.001$).

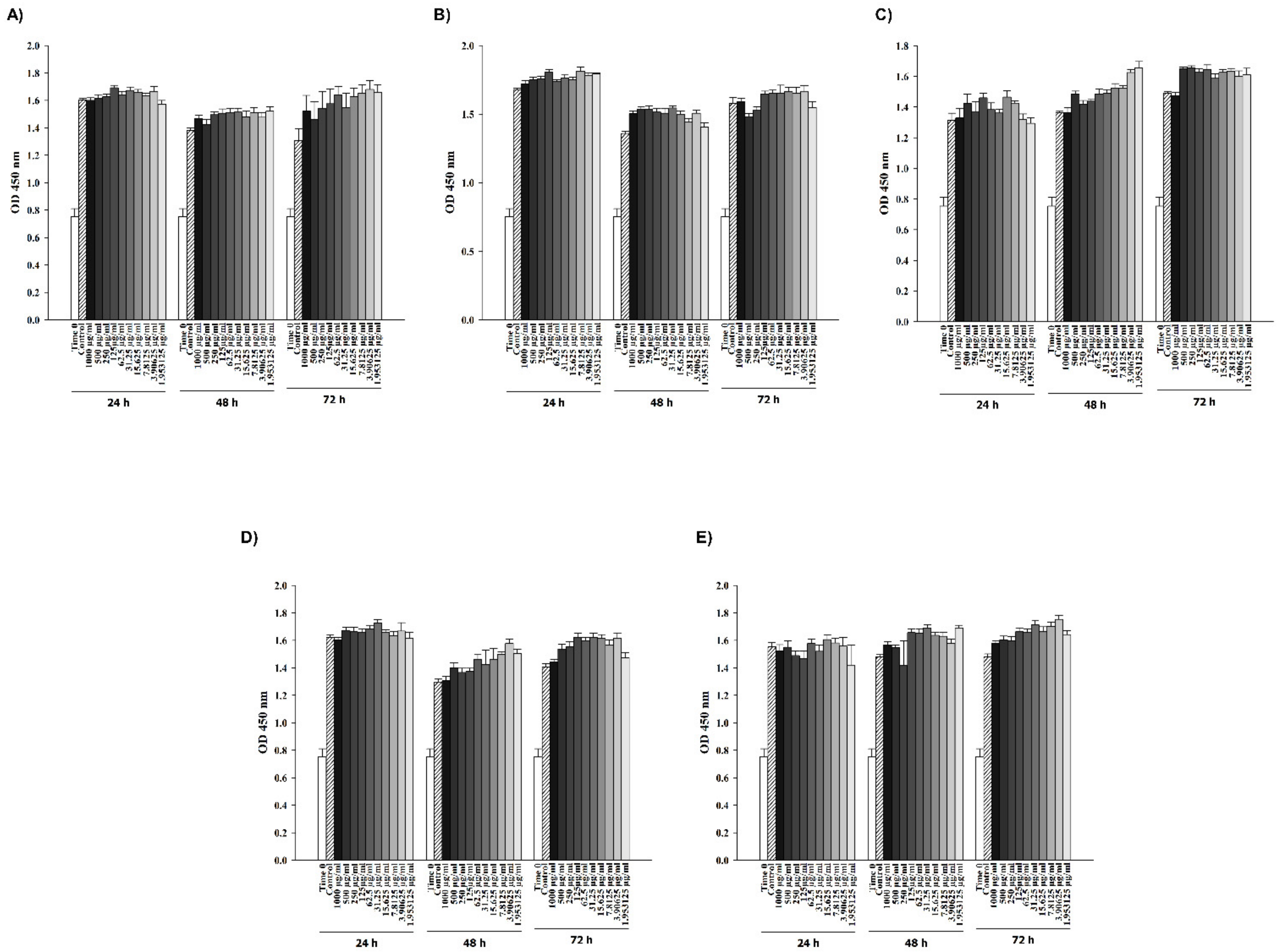

Figure S18: Effects of *A. pisidicus* water extract on cell viability in 293T lines. Cell viability was assessed by WST-1 assays and the results are presented as optical density (OD_{450}) values at 450 nm for A) Fw, (B) Lw, (C) Rw, (D) Sw and (E) WPw for 24, 48 and 72 h (* $p < 0.05$, ** $p < 0.01$ and *** $p < 0.001$).
